# Cold and heterogeneous T cell repertoire is associated with copy number aberrations and loss of immune genes in small-cell lung cancer

**DOI:** 10.1038/s41467-021-26821-8

**Published:** 2021-11-17

**Authors:** Ming Chen, Runzhe Chen, Ying Jin, Jun Li, Xin Hu, Jiexin Zhang, Junya Fujimoto, Shawna M. Hubert, Carl M. Gay, Bo Zhu, Yanhua Tian, Nicholas McGranahan, Won-Chul Lee, Julie George, Xiao Hu, Yamei Chen, Meijuan Wu, Carmen Behrens, Chi-Wan Chow, Hoa H. N. Pham, Junya Fukuoka, Jia Wu, Edwin Roger Parra, Latasha D. Little, Curtis Gumbs, Xingzhi Song, Chang-Jiun Wu, Lixia Diao, Qi Wang, Robert Cardnell, Jianhua Zhang, Jing Wang, Xiuning Le, Don L. Gibbons, John V. Heymach, J. Jack Lee, William N. William, Chao Cheng, Bonnie Glisson, Ignacio Wistuba, P. Andrew Futreal, Roman K. Thomas, Alexandre Reuben, Lauren A. Byers, Jianjun Zhang

**Affiliations:** 1 Department of Radiation Oncology, Sun Yat-sen University Cancer Center, State Key Laboratory of Oncology in South China, Collaborative Innovation Center for Cancer Medicine, Sun Yat-sen University, Guangzhou, Guangdong 510060 China; 2 The Cancer Hospital of the University of Chinese Academy of Sciences (Zhejiang Cancer Hospital), Hangzhou, Zhejiang 310022 China; 3 Institute of Basic Medicine and Cancer (IBMC), Chinese Academy of Sciences, Hangzhou, Zhejiang 310022 China; 4 Zhejiang Key Laboratory of Radiation Oncology, Hangzhou, Zhejiang 310022 China; 5 Department of Thoracic/Head and Neck Medical Oncology, the University of Texas MD Anderson Cancer Center, Houston, Texas 77030 USA; 6 Department of Genomic Medicine, the University of Texas MD Anderson Cancer Center, Houston, Texas 77030 USA; 7 Department of Bioinformatics and Computational Biology, the University of Texas MD Anderson Cancer Center, Houston, Texas 77030 USA; 8 Department of Translational Molecular Pathology, the University of Texas MD Anderson Cancer Center, Houston, Texas 77030 USA; 9 Cancer Research United Kingdom-University College London Lung Cancer Centre of Excellence, London, WC1E6BT UK; 10 Department of Translational Genomics, Faculty of Medicine and University Hospital Cologne, University of Cologne, Cologne, 50931 Germany; 11 Department of Otorhinolaryngology, Head and Neck Surgery, University Hospital Cologne, 50937 Cologne, Germany; 12 Department of Pathology, Nagasaki University Graduate school of Biomedical Sciences, Nagasaki, Japan; 13 Department of Image Physics, the University of Texas MD Anderson Cancer Center, Houston, Texas 77030 USA; 14 Department of Biostatistics, the University of Texas MD Anderson Cancer Center, Houston, Texas 77030 USA; 15 Institute for Clinical and Translational Research, Baylor College of Medicine, Houston, Texas 77030 USA; 16 Department of Translational Genomics, Medical Faculty, University of Cologne, Cologne, 50931 Germany; 17 Department of Pathology, Medical Faculty, University Hospital Cologne, Cologne, 50931 Germany; 18 DKFZ, German Cancer Research Center and German Cancer Consortium (DKTK), Heidelberg, 69115 Germany

**Keywords:** Tumour immunology, Cancer genomics, Small-cell lung cancer, Tumour heterogeneity

## Abstract

Small-cell lung cancer (SCLC) is speculated to harbor complex genomic intratumor heterogeneity (ITH) associated with high recurrence rate and suboptimal response to immunotherapy. Here, using multi-region whole exome/T cell receptor (TCR) sequencing as well as immunohistochemistry, we reveal a rather homogeneous mutational landscape but extremely cold and heterogeneous TCR repertoire in limited-stage SCLC tumors (LS-SCLCs). Compared to localized non-small cell lung cancers, LS-SCLCs have similar predicted neoantigen burden and genomic ITH, but significantly colder and more heterogeneous TCR repertoire associated with higher chromosomal copy number aberration (CNA) burden. Furthermore, copy number loss of IFN-γ pathway genes is frequently observed and positively correlates with CNA burden. Higher mutational burden, higher T cell infiltration and positive PD-L1 expression are associated with longer overall survival (OS), while higher CNA burden is associated with shorter OS in patients with LS-SCLC.

## Abstract (web summary)

Small-cell lung cancer (SCLC) is an aggressive disease with limited therapeutic options. Here the authors perform an immunogenomic analysis of limited-stage SCLC, revealing a homogeneous mutational landscape, but limited T-cell infiltration and a cold and heterogeneous T cell repertoire.

## Introduction

Small-cell lung cancer (SCLC) accounts for ~15% of all newly diagnosed lung cancers, leading to ~30,000 deaths in the United States annually^1^. SCLC is a highly aggressive cancer characterized by rapid growth and high rates of early local and distant metastases^2,3^. At initial diagnosis, around one-third of SCLC patients present with cancer confined to one hemithorax, defined as limited-stage disease (LS) that can be treated with chemotherapy combined with radiotherapy or surgical resection, while the remaining patients present with extensive-stage disease (ES) exhibiting extensive lymph node involvement and/or distant metastases usually treated with palliative chemotherapy with or without immune checkpoint blockade (ICB) therapy^4–6^. Although most SCLC patients experience an initial response, nearly all patients recur with rapidly progressing disease resistant to late-line treatments. Despite extensive research, only modest advances have been achieved in the treatment of SCLC over the past 30 years with median survival less than a year and 5-year overall survival (OS) below 7% for ES-SCLC^1,7,8^. Recently, the addition of ICB to chemotherapy has become a new standard of care for advanced SCLC, although it confers only an improvement of 2–3 months in survival^6^. The National Cancer Institute (NCI) has identified SCLC as a recalcitrant malignancy^1^. Translational studies to understand the mechanisms underlying recurrence and therapeutic resistance remain an unmet need to design novel therapeutic strategies.

Tumors are composed of cancer cells and stromal cells of distinct molecular and phenotypic features, a phenomenon termed intratumor heterogeneity (ITH). Evolutionary theory suggests that populations of high genetic variation have survival advantages^9^. Similarly, tumors of complex ITH may be difficult to eradicate. Higher levels of molecular ITH have been demonstrated to associate with inferior outcome of cancer patients^10–12^. We and others have previously delineated the ITH architecture of non-small cell lung cancers (NSCLCs) at genomic, epigenetic and gene expression levels utilizing multiregional sequencing and demonstrated that complex ITH was associated with inferior survival^10,12–17^. It has been speculated that SCLC has an extremely complex ITH architecture that leads to poor prognosis^18^. Another plausible explanation for the poor outcome is that SCLC is associated with an immunosuppressive tumor microenvironment, particularly T cell responses^19,20^. In localized NSCLC, our recent work has revealed that a cold and heterogeneous T cell receptor (TCR) repertoire is associated with inferior survival^13,21^. The genomic and TCR ITH architecture of SCLC and their potential clinical impact have not been well studied, largely due to lack of adequate tumor specimens^22–25^ as surgical resection is not the standard of care for the majority of SCLC patients.

In this study, through international collaboration, we conduct multi-region whole-exome sequencing (WES) and TCR sequencing of 50 tumor samples from 19 resected LS-SCLCs to depict the immunogenomic ITH architecture of SCLC and compare these LS-SCLCs with localized NSCLCs and assess the impact of immunogenomic attributes on patient survival. We demonstrate that despite a homogeneous genomic landscape, SCLC exhibits a cold and heterogeneous T-cell infiltration associated with higher chromosomal copy number aberration (CNA) burden and loss of essential immune genes, potentially leading to ineffective antitumor immune surveillance and inferior survival.

## Results

### Overall homogeneous genomic landscape in SCLC

A total of 50 spatially separated tumor regions (hereafter referred to as region) from 19 resected LS-SCLC tumors (hereafter referred to as tumor) with adequate tissue available were subjected to WES (Supplementary Fig. 1, Supplementary Fig. 2, Supplementary Data 1). All patients underwent upfront surgery without preoperative chemotherapy or radiation therapy. In total, 3773 nonsilent (nonsynonymous, frameshift, stop-gain, and stop-loss) mutations were identified from these 50 tumor regions (Supplementary Data 2) with a median nonsilent tumor mutational burden (TMB) of 4.69/Mb (interquartile (25th–75th) range: 2.66–6.43/Mb). TMB varied substantially between patients, but was similar between different regions within the same tumors (Supplementary Fig. 3).

**Fig. 1:**
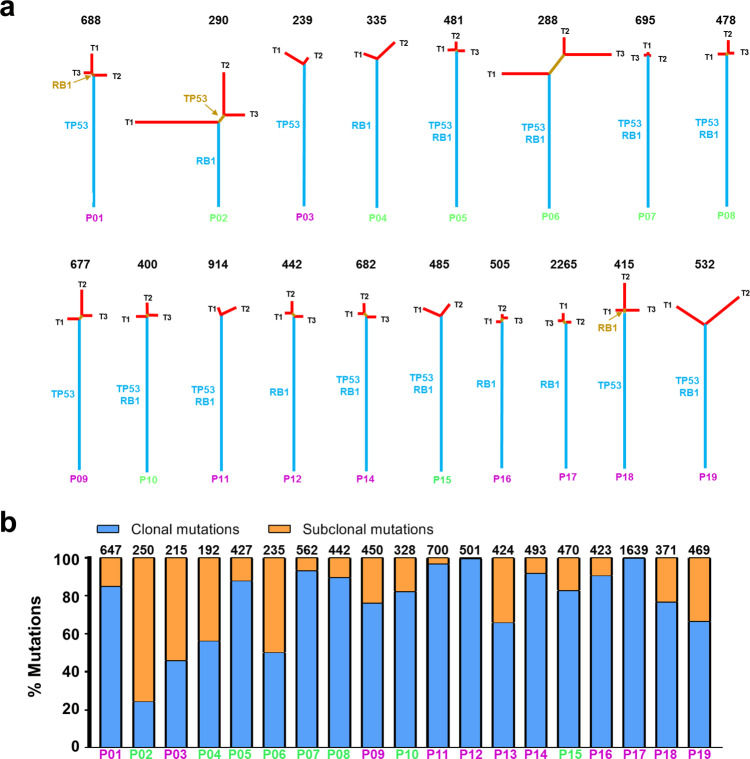
Genomic intratumor heterogeneity (ITH) and clonal architecture of limited-stage small-cell lung cancers (LS-SCLCs). **a** Phylogenetic trees of 18 limited-stage SCLC tumors (LS-SCLCs) with multi-region whole-exome sequencing (WES). Blue, brown and red lines represent trunk, branch, and private mutations, respectively. The length of trunk (blue), branch (brown), and private branch (red) is proportional to the numbers of mutations shared by 3, 2, or 1 tumor regions. The total number of mutations is listed above the phylogenetic tree of each tumor. TP53 and RB1 mutations are mapped to the phylogenetic trees as indicated. **b** Global clonal architecture of SCLC at tumor level. PyClone was run on merged bam files from different regions of the same tumors. Mutations were classified as clonal (present in the cluster with the highest cellular prevalence, blue) or subclonal (orange) in each tumor. The total number of mutations in each tumor is listed on the top of each bar. Note: the numbers of mutations in each tumor are less than those in phylogenetic analysis as the clonal status of some mutations could not be inferred by PyClone. Patient ID: purple = alive; green = deceased. Source data are provided as a Source Data file.

We constructed phylogenetic trees of 18 SCLCs for which multi-region WES data were available (P13 only had one tumor region and was excluded from this analysis) to depict the genomic ITH and the evolutionary trajectory of these SCLCs^12^. A median of 80.1% (28–93%) (interquartile (25th–75th) range: 72–84%) of mutations were mapped to the trunks of these 18 SCLCs (Fig. 1a) representing ubiquitous mutations present in all regions within the same tumors, compared with 72% (8–99.6%) (interquartile (25th–75th) range: 55–84%) trunk mutations in 100 early-stage NSCLC in TRACERx cohort (*p* = 0.218)^14^. Considering that the number of regions per tumor may introduce bias into the proportion of trunk mutations, we calculated the mutational Jaccard index (JI) between each pair of specimens within the same tumors that is not influenced by number of regions (Supplementary Fig. 4) in 18 SCLCs (Supplementary Fig. 5a). The results demonstrated that genomic JI was comparable in the cohort of SCLC and NSCLC from the TRACERx cohort (Supplementary Fig. 5b, 0.83 vs. 0.82, *p* = 0.6058).

Next, we assessed the chromosomal copy number aberrations (CNAs) in this cohort of LS-SCLC using a gene-based CNA analysis algorithm^26^ for WES data that compares the CNA burden between different samples (Supplementary Fig. 6). The CNA burden was similar between different regions within the same tumors, but varied substantially between patients (Supplementary Fig. 7a). The median CNA JI was 0.50 (range: 0.02–0.93) (interquartile (25th–75th) range: 0.27–0.85, Supplementary Fig. 5c), higher than that of NSCLC tumors from the TRACERx cohort (median: 0.21, range: 0–0.99) (interquartile (25th–75th) range: 0.06–0.35, Supplementary Fig. 5d). CNA heterogeneity varied at different chromosomal locations (Supplementary Fig. 7b).

To further understand the global clonal architecture of SCLC tumors, we merged WES data from different regions within the same tumors and calculated the percent clonal and subclonal mutations at tumor level by Pyclone^27^. The results revealed a median of 82.8% (24.4–99.8%) (interquartile (25th–75th) range: 65.8–91.7%) of clonal mutations at global tumor level (Fig. 1b), once again suggesting a relatively homogeneous mutational landscape.

A total of 134 cancer gene alterations were identified in these tumors (Supplementary Fig. 8). As expected, the most frequently altered cancer genes were RB1 and TP53, identified in 16 (84%) and 15 (79%) of the 19 patients, respectively. Importantly, 86 of 134 (64%) cancer gene alterations were detected in all regions within the same tumors and 30 of 34 (88%) cancer gene point mutations (with known clonal status at tumor level) were clonal. These results suggest that these cancer gene alterations were early genomic events during evolution of SCLC and the genomic landscape of these SCLC tumors was homogeneous not only quantitatively, but also qualitatively in key cancer gene alterations.

### Different mutational processes are associated with early versus late mutagenesis in SCLC

Understanding how mutational processes shape cancer evolution may inform the mechanisms underlying tumor adaptation^28^. We analyzed the mutational spectrum and signatures in these SCLCs^29^. C > A transversions were the most common nucleotide substitutions (Supplementary Fig. 9) and Cosmic Signature 4 (associated with cigarette smoking) was the predominant mutational signature (Supplementary Fig. 10a) as expected, given that all 19 patients were either smokers or heavy second-hand smokers.

To further dissect the mutational processes associated with early versus later carcinogenesis of these SCLC tumors, we delineated the mutational signatures of trunk mutations representing early genomic events and nontrunk mutations representing later events, respectively^28^. As shown in Supplementary Fig. 10b, c, among the top 5 mutational signatures, Cosmic Signature 4 remained as the predominant signature in trunk mutations contributing to 62% of the top 5 signatures, consistent with previous reports that smoking-associated mutational processes play critical roles during early mutagenesis of lung cancers^12,14,30^. On the other hand, the contribution of Signature 4 was significantly reduced (29% for nontrunk mutational signatures versus 62% for trunk mutational signatures among the top 5 signatures, *p* = 0.002), while Cosmic Signature 3 (associated with defect of DNA double-strand break repair) emerged as the predominant signature for nontrunk mutations (45% for nontrunk mutational signatures versus 6% for trunk mutational signatures among the top 5 signatures, *p* < 0.0001). These results highlight the dynamic nature of mutagenesis at different time points during evolution of SCLC and suggest that smoking-associated mutational processes play essential roles during early carcinogenesis of this cohort of SCLC, while later evolution may be associated with other mutational processes such as DNA repair defects.

### Cold TCR repertoire in SCLC tumors

**Fig. 2:**
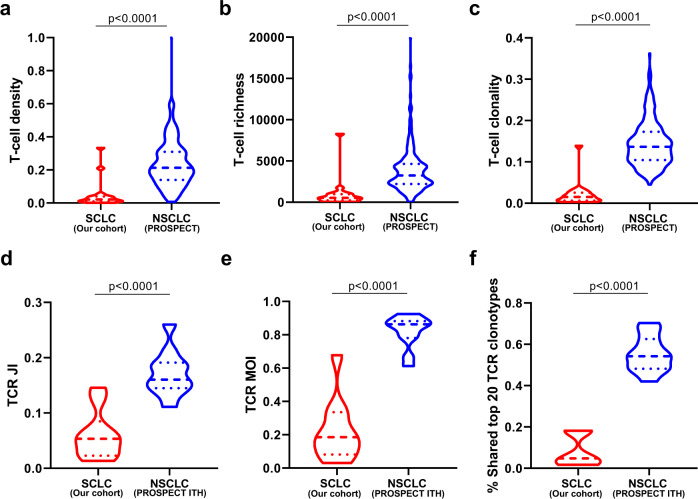
TCR metrics in small-cell lung cancer (SCLC) versus non-small cell lung-cancer (NSCLC) tumors (the PROSPECT cohort). **a** T-cell density—an estimate of the proportion of T cells in a specimen, **b** T-cell richness—a measure of T-cell diversity and (**c**) T-cell clonality—a metric indicating T-cell expansion and reactivity, were derived from 19 SCLCs (red) *versus* 236 NSCLCs (blue) from the PROSPECT cohort. TCR intratumor heterogeneity (ITH) in 10 SCLC *versus* 11 NSCLC using (**d**) the average Jaccard index (JI), a metric representing the proportion of shared T-cell clonotypes, **e** Morisita index (MOI), a metric taking into consideration not only the composition of T-cell clonotypes but also the abundance of individual T-cell clonotypes and (**f**) proportion of shared top 20 TCR clonotypes between any paired samples within the same tumors in SCLC (blue) versus NSCLC tumors (red) with multiregional TCR data available. The difference of TCR metrics between SCLC and NSCLC was evaluated using two-sided Mann–Whitney test. Source data are provided as a Source Data file.

We next performed TCR sequencing in 36 tumor specimens (1–3 regions per tumor) and 16 tumor-adjacent lung tissues from patients with adequate DNA remaining. T-cell density, an estimate of the proportion of T cells in a specimen, ranged from 0.11% to 33% with a median of 1.7% (interquartile (25th–75th) range: 0.57–3.97%) (Supplementary Fig. 11a). T-cell richness, a measure of T-cell diversity, ranged from 38 to 8286 unique T cells (median: 510) (interquartile (25th–75th) range: 181–1034) per specimen (Supplementary Fig. 11b) and T-cell clonality, a metric depicting T-cell expansion and reactivity, ranged from 0.002 to 0.139 (median = 0.009) (interquartile (25th–75th) range: 0.005–0.0025) (Supplementary Fig. 11c). Compared with tumor-adjacent lung tissues (≥2 cm from tumor margin), SCLC tumors demonstrated lower T-cell density, richness, and clonality (*p* = 0.0580, *p* = 0.0067, and *p* = 0.0166, respectively, Supplementary Fig. 11d–f), indicating a reduced T-cell infiltration, diversity, and proliferation in tumor tissues similar to NSCLC^21^. As shown in Supplementary Fig. 11, P13 was an outlier in TCR parameters. We therefore re-ran this analysis excluding P13, which did not lead to significant changes (Supplementary Fig. 12). Of particular interest, compared with the TCR repertoire data generated using the same method from NSCLCs (the PROSPECT cohort), all three TCR metrics were significantly lower than those from NSCLCs (T-cell density 0.014 versus 0.21, *p* < 0.0001 (Fig. 2a); diversity 510 versus 3246, *p* < 0.0001 (Fig. 2b); and clonality 0.009 versus 0.14, *p* < 0.0001 (Fig. 2c))^21^. Similarly, the total TCR templates were also significantly lower in SCLC tumors compared with NSCLC tumors (Supplementary Fig. 13).

**Fig. 3:**
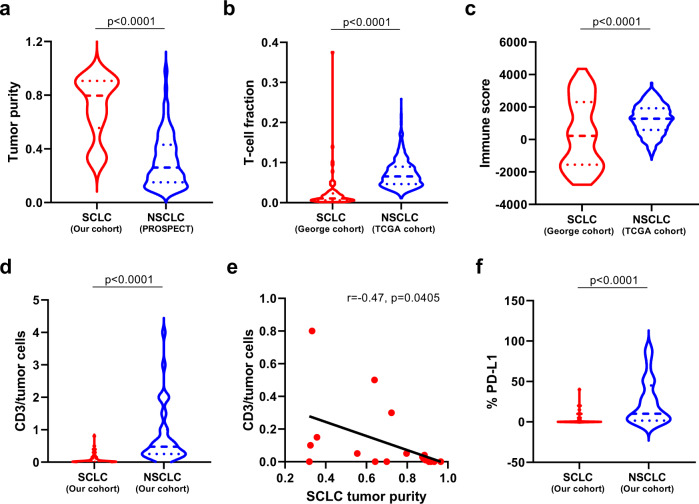
Comparison of immune features in small-cell lung cancer (SCLC) versus non-small cell lung cancer (NSCLC). **a** Tumor purity in SCLCs versus NSCLCs. Tumor purity was derived from whole-exome sequencing (WES) data from 19 SCLC tumors (blue) versus 242 NSCLC tumors (red) from the PROSPECT cohort. **b** T-cell infiltration in SCLCs compared with NSCLCs. T-cell infiltration was derived by deconvolution of RNA sequencing (RNA-seq) data of 81 SCLC tumors (George cohort) versus 1027 NSCLC tumors from TCGA. **c** Immune score in SCLCs compared with NSCLCs. The immune score was calculated from RNA-seq data to quantify all immune cells within the tumors from 81 SCLC tumors (George cohort) versus 1027 NSCLC tumors from TCGA. **d** CD3 + tumor-infiltrating lymphocytes (TILs) of SCLC (*n* = 67) versus NSCLC (*n* = 68) tumors by immunohistochemistry (IHC). The y axis represents CD3 + TILs: tumor-cell ratio. **e** Association of tumor purity with CD3 + TILs in SCLCs (*n* = 19). The y axis represents CD3 + TILs: tumor-cell ratio. **f** Expression of programmed death ligand-1 (PD-L1) by IHC in SCLC (*n* = 67) versus NSCLC (*n* = 68) tumors. The difference of immune features between SCLC and NSCLC was evaluated using two-sided Mann–Whitney test. The correlation coefficient (r) of tumor purity with CD3 + TILs was assessed by two-tailed Spearman’s rank-correlation test. Source data are provided as a Source Data file.

Higher levels of immune-cell infiltration can lead to low tumor purity. Vice versa, lower tumor purity can indicate a higher level of immune-cell infiltration. We estimated tumor purity by Sequenza^31^ in this cohort of LS-SCLCs and compared that with localized NSCLCs from the PROSPECT cohort. As shown in Fig. 3a, significantly higher tumor purity was observed in these SCLC compared with NSCLC from the PROSPECT cohort supporting possible lower immune infiltration in SCLC.

Furthermore, we derived the immune score by quantifying the density of all immune cells within tumors and estimated the fraction of T-cell infiltration by deconvoluting previously published RNA sequencing (RNA-seq) data of 81 SCLCs^23^ and compared those with 1027 NSCLCs from TCGA^30,32^. In line with TCR repertoire findings, both immune score and estimated T-cell fraction were significantly lower in SCLCs than NSCLCs (*p* < 0.0001, *p* < 0.0001, respectively, Fig. 3b, c).

To orthogonally assess tumor-infiltrated T cells, we performed immunohistochemistry (IHC) by T-cell marker CD3 and programmed death ligand-1 (PD-L1) (Supplementary Fig. 14) on 66 SCLC tumors, including 18/19 tumors in the current study (one tumor tissue was exhausted) and 68 NSCLC tumors (Supplementary Data 1) with matched clinical characteristics, including sex, age, smoking status and tumor size (Table 1). As shown in Fig. 3d, SCLC tumors had significantly lower T-cell infiltration than NSCLC tumors (p < 0.0001). Importantly, the T-cell infiltration was negatively associated with tumor purity (*r* = −0.47, *p* = 0.0405, Fig. 3e), indicating lower T-cell infiltration was one potential reason underlying observed higher tumor purity in SCLCs than NSCLCs. Moreover, the PD-L1 expression was also significantly lower in SCLCs than NSCLCs (*p* < 0.0001, Fig. 3f). Taken together, these data suggest that SCLC had an overall lower T-cell infiltration rather than higher level of adaptive immunosuppression compared with NSCLCs at the time of resection.

**Table 1:** Clinical characteristic comparison of SCLC and NSCLC patients.

	SCLC (*n* = 67)	NSCLC (*n* = 68)	*p* value
Age (median) (yrs)	64 (38–83)	64 (36–79)	0.9659
Gender			0.155
Female	11	18	
Male	56	50	
Smoking status			0.2025
Never smokers	16	23	
Smokers	51	45	
Tumor size (median) (cm)	2.8 (1–10)	3 (1–8.5)	0.6205
TNM stage			0.0666
I	30	31	
II	23	13	
III	14	24	

The *p* values were calculated by two-sided Mann–Whitney test for age, Chi-squared test for gender, Chi-squared test for smoking status and Mann–Whitney test for tumor size.

### Substantial TCR repertoire heterogeneity in SCLC

**Fig. 4:**
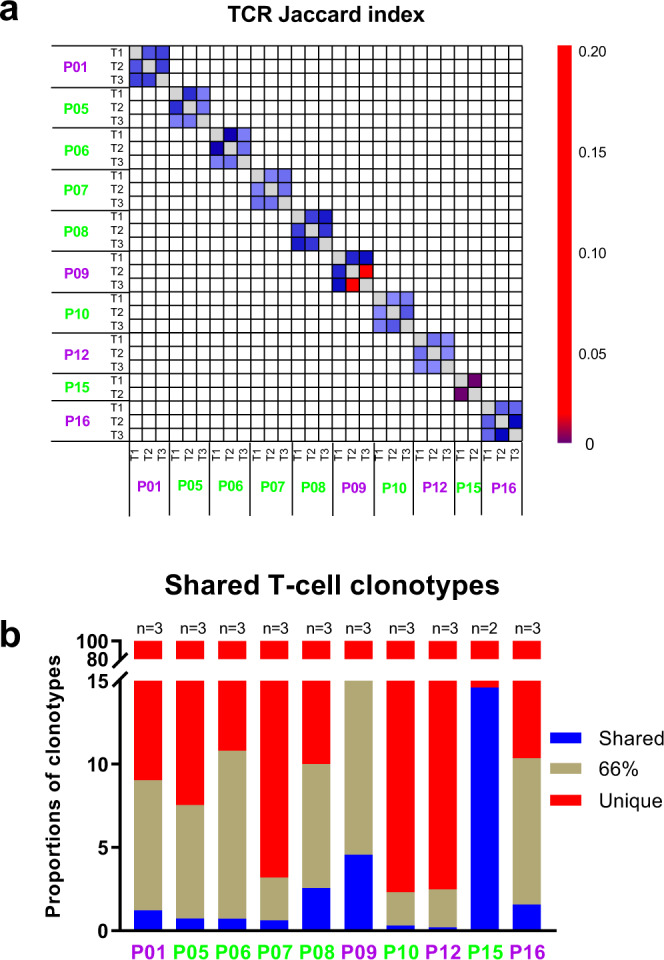
Substantial T cell receptor (TCR) repertoire intratumor heterogeneity (ITH) in small-cell lung cancer (SCLC). **a** Quantification of TCR ITH by Jaccard index (JI), a metric representing the proportion of shared T-cell clonotypes between two samples in 10 SCLC patients with multiregion TCR sequencing data. **b** Proportions of T-cell clonotypes detected in all regions (shared, blue), in 2/3 (brown) and restricted to a single region (red) from the same tumors in 10 SCLC patients with multiregion TCR sequencing data. Patient ID: purple = alive; green = deceased. Source data are provided as a Source Data file.

To gain further insights into TCR heterogeneity, we calculated the JI measuring the proportion of shared T-cell clonotypes between two samples. Substantial TCR heterogeneity was evident across all SCLC tumors, with a median JI of 0.05 (0.02–0.15) in the 10 SCLCs with multiregion TCR data available (Fig. 4a), significantly lower than the 11 localized NSCLCs^13^ (median 0.05 in SCLC vs. 0.16 in NSCLC, *p* < 0.0001) (Fig. 2d) with multiregion TCR data available^13^. Furthermore, 79.9–97.7% of T-cell clones were restricted to individual tumor regions, while only 0.2–14.6% (median: 0.98%, interquartile (25th–75th) range: 0.54–3.06%) were identified in all regions within the same tumors (Fig. 4b), significantly lower than NSCLC (median: 5.7%, range: 1.6–14.5%) (interquartile (25th–75th) range: 3.6–7.4%) (*p* = 0.0048)^13^ demonstrating profound TCR ITH in SCLC even beyond NSCLC. Of note, TCR JI was positively correlated with T-cell density, richness and clonality, although not universally statistically significant (Supplementary Fig. 15a–c).

To compare shared TCR clonotypes considering T-cell expansion, we used two additional metrics: Morisita index (MOI), which takes into consideration not only T-cell clonotype composition but also abundance, and the proportion of shared top (most expanded/abundant) 20 clonotypes. Similar to TCR JI, compared with the NSCLCs^13^, SCLC tumors showed significantly lower TCR MOI and shared top 20 TCR clonotypes (Fig. 2e, f), suggesting a higher level of TCR ITH in SCLC tumors for not only the overall TCR repertoire but also for the most expanded/active T cells.

### Frequent copy number aberrations, loss of IFN-γ pathway genes, and HLA LOH in SCLCs

To identify the genomic aberrations underlying a cold TCR repertoire in SCLC, we first assessed (HLA)-A-, -B-, and -C-presented neoantigens in silico^33,34^. A median of 88 (39–503) (interquartile (25th–75th) range: 53–125) predicted neoantigens (IC_50_ < 500 nmol/L) per tumor were detected (Supplementary Fig. 16a), which was similar to NSCLCs from the PROSPECT cohort (median: 72/tumor, 2–801, interquartile (25th–75th) range: 35–125, p = 0.25). Similar to somatic mutations, 81% (48–93%, interquartile (25th–75th) range: 72–89%) of predicted neoantigens were present across different regions within the same tumors (Supplementary Fig. 16b), and 91% of predicted neoantigens (21–100%, interquartile (25^th^–75^th^) range: 77–82%) were associated with clonal mutations at the tumor level. These results suggest that the cold TCR repertoire in SCLC is unlikely to be due to a low clonal neoantigen burden.

**Fig. 5:**
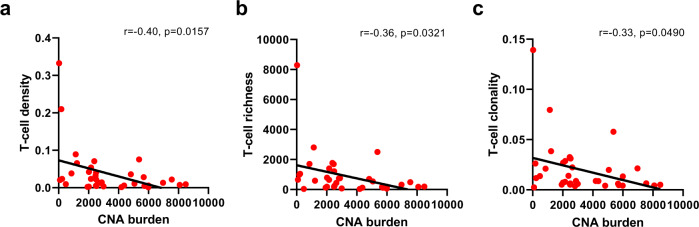
Associations of chromosomal copy number aberrations (CNAs) with T cell receptor (TCR) repertoire. CNA burden and its negative correlations with T-cell (**a**) density, (**b**) richness, and (**c**) clonality in 36 small-cell lung cancer (SCLC) samples with both CNA and TCR data available. The correlation coefficient (r) was assessed by two-tailed Spearman’s rank-correlation test. Source data are provided as a Source Data file.

We next assessed the associations between CNA burden and TCR repertoire^35,36^. A median of 2310 CNA events per tumor (26–8044, interquartile (25th–75th) range: 444–4318) were identified from these SCLCs (Supplementary Fig. 7a), significantly higher than 111 per tumor (range: 0–7741, interquartile (25th–75th) range: 0–561) in NSCLCs from the PROSPECT cohort (*p* < 0.0001)^21^, which have exhibited a significantly more active TCR repertoire than this cohort of SCLCs (Fig. 2a–c). Importantly, the CNA burden was negatively associated with T-cell density, richness, and clonality in this cohort of SCLCs (Fig. 5a–c, *r* = −0.40, *p* = 0.0157; *r* = −0.36, *p* = 0.0321; *r* = −0.33, *p* = 0.0490, respectively), suggesting that higher CNA burden was an important genomic feature associated with an impaired TCR repertoire in these SCLCs.

One potential impact of CNA on immune evasion is loss of neoantigens^35,37^. In this cohort, predicted neoantigen-associated genes were not observed to be more frequently lost than other mutated genes (Supplementary Fig. 17a). We next explored whether loss of heterozygosity (LOH) is associated with predicted neoantigen loss. As shown in Supplementary Fig. 17d, LOH was enriched in chromosomal regions 3p, 5, 13, 15, and 17p, consistent with previous reports^23,38^. Copy number neutral LOH (CNN–LOH) was more common in predicted neoantigen-associated genes than mutated genes not associated with neoantigens (Supplementary Fig. 17b). The same trend was observed in copy number loss LOH (CNL–LOH), though the difference was not statistically significant (Supplementary Fig. 17c). These results suggest that neoantigen loss through LOH could be a potential mechanism underlying immune evasion in this cohort of SCLCs. An important follow-up question is whether these predicted neoantigens were maintained or lost under LOH. Presumably, all LOH events associated with neoantigens must be subclonal, otherwise, these mutations leading to neoantigens would not have been detected. If these predicted neoantigens are kept under (or occurred after) LOH, it could indicate either a lack of immune evasion or inability of LOH as a mechanism of immune evasion. As there are currently no appropriate computational approaches to reliably infer the clonal status of LOH, we used trunk (LOH present in all regions within the same tumors) versus branch (LOH present in some but not all regions within the same tumors) status to represent the clonal status of LOH. Overall, a median of 89.8% (ranging from 40.2% to 99.3%) of LOH events were trunk events shared by all regions within the same tumors (Supplementary Fig. 18 and 19), suggesting that these LOH events were early molecular events during the evolution of these SCLC tumors. Importantly, only a median of 16% (range: 1.5–26%) of genes associated with predicted neoantigens showed evidence of LOH and a median of 18% (range: 0–73%) of LOH events associated with predicted neoantigens were branched albeit numerically higher when all LOH events were considered (Supplementary Fig. 20). With all the technical caveats acknowledged, these results did not support LOH as a major mechanism underlying immune evasion in this cohort of SCLC tumors.

**Fig. 6:**
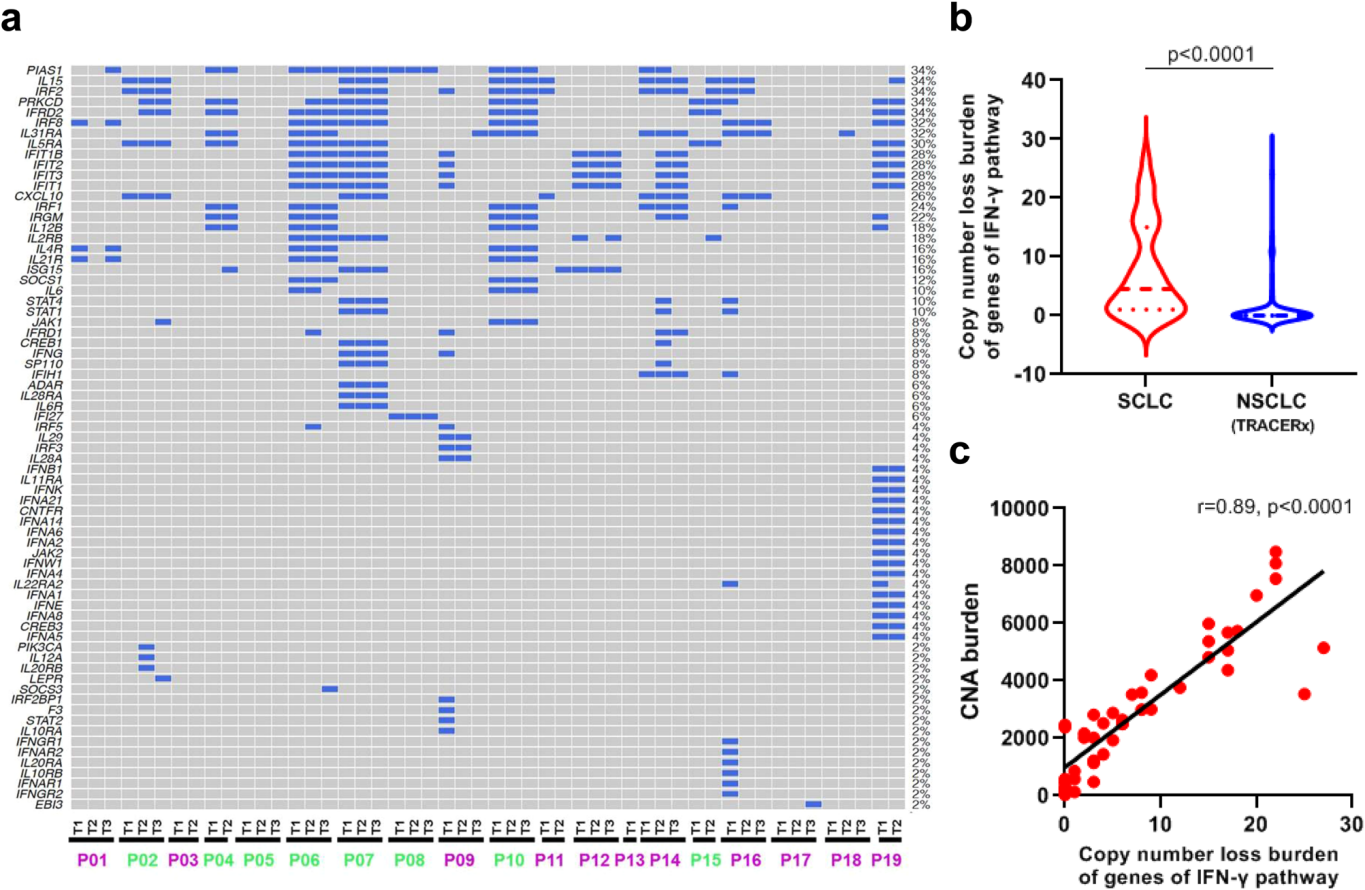
Copy number loss of interferon gamma (IFN-γ) pathway genes in small-cell lung cancer (SCLC) and comparison with non-small cell lung-cancer (NSCLC) tumors. **a** Copy number loss of IFN-γ pathway genes in 50 SCLC samples from 19 SCLC patients. Purple patient IDs = alive; Green patient IDs = deceased. **b** Copy-number loss burden of IFN-γ pathway genes in 50 SCLC samples (blue) versus 327 NSCLC samples (red) from TRACERx. **c** Correlation of copy number alteration (CNA) burden of IFN-γ pathway genes with overall CNA burden in SCLC (*n* = 50). The difference of copy number loss burden of IFN-γ pathway genes between SCLC and NSCLC was evaluated using two-sided Mann–Whitney test. The correlation coefficient (r) of CNA burden of IFN-γ pathway genes with overall CNA burden was assessed by two-tailed Spearman’s rank-correlation test. Source data are provided as a Source Data file.

Mutations of essential genes (e.g., B2M, JAK1, and JAK2) of antigen-presentation (APC) pathways and copy number loss of interferon gamma (IFN-γ) pathway genes have been reported to play pivotal roles in immune evasion^39,40^. In this cohort of SCLCs, we did not detect any mutations in APC-pathway genes. However, copy-number loss of IFN-γ pathway genes was frequently observed in this cohort of SCLCs (Fig. 6a) and was significantly more common than NSCLCs (Fig. 6b). Importantly, copy-number loss in IFN-γ pathway genes was positively associated with the CNA burden (Fig. 6c). These data suggest that frequent loss of essential immune genes associated with the high global CNA burden may be one potential genomic basis underlying immune evasion in SCLCs.

LOH of HLA has also been reported as a potential immune-evasion mechanism across different cancers^41,42^. Evidence of HLA LOH was revealed in 9 of 19 SCLC tumors, numerically higher than in NSCLCs from the TRACERx (9/19 versus. 36/90, *p* = 0.61) and the PROSPECT cohort (9/19 versus 60/216, *p* = 0.11), although the difference did not reach statistical significance likely due to the small sample size. Among the 21 samples with HLA LOH in this cohort of SCLC, a median of 52.7% (0–89.7%) of predicted neoantigens bound to the lost allele (Supplementary Fig. 21) supporting HLA LOH as one potential mechanism underlying immune evasion. On the other hand, some tumors (P01, P04, and P14 for example) had only a small proportion of predicted neoantigens binding to the lost allele, suggesting that other factors could have contributed to immune evasion in these tumors.

Taken together, these results suggest that there was once immune pressure during carcinogenesis of these SCLC. Loss of essential immune genes and HLA LOH could be common genomic alterations underlying immune evasion. Eventually, these SCLC tumors were able to survive immune pressure, leading to a limited immune infiltration, as evidenced by low T-cell infiltration and low T-cell expansion. Interestingly, among the 9 SCLC tumors, 5 tumors had HLA LOH in all regions (P04, 09, 17, 18 and 19), while four tumors had HLA LOH in 1 or 2 regions (P01, 02, 07 and 14). Similarly, loss of different IFN-γ pathway genes was observed in different regions of the same SCLC tumors (Fig. 6a). These data indicate that distinct immune-escape mechanisms could be at play not only in different patients but also in different subclones within the same tumors.

### Genomic and TCR ITH associated with survival in patients with SCLC

**Fig. 7:**
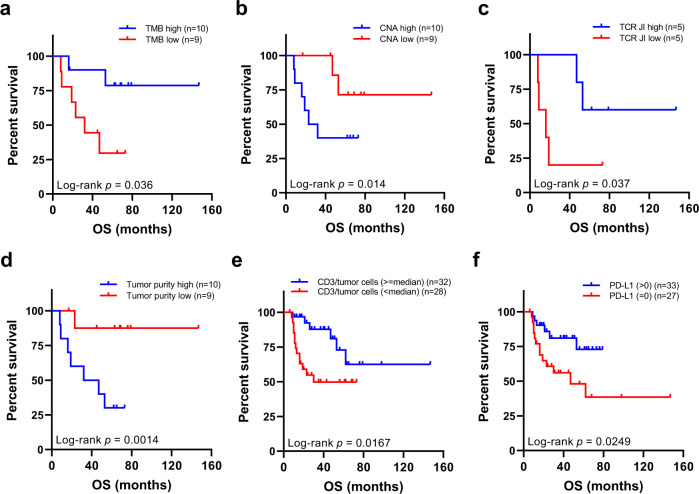
Association of overall survival (OS) with immunogenomic landscape. **a** OS of patients with higher (above median, blue) tumor mutational burden (TMB) versus patients with lower (below median, red) TMB. **b** OS of patients with higher (above median, blue) copy number aberration (CNA) burden versus patients with lower (below median, red) CNA burden. **c** OS of patients with more homogeneous T-cell receptor (TCR) repertoire (higher above-median TCR Jaccard index (JI), blue) versus patients with more heterogeneous TCR repertoire (lower below-median TCR JI, red). **d** OS of patients with tumors of higher (above median, blue) tumor purity versus patients with tumors of lower (below median, red) tumor purity. **e** OS of patients with tumors of higher (no less than median, blue) CD3 + tumor-infiltrating lymphocytes (TILs) versus patients with tumors of lower (below median, red) CD3 + TILs. **f** OS of patients with tumors of positive (above 0, blue) programmed death ligand-1 (PD-L1) expression versus patients with tumors of negative (equal to 0, red) PD-L1 expression. Two-sided log-rank test was used for survival analysis. Source data are provided as a Source Data file.

We next attempted to assess whether genomic and T-cell features impact clinical outcome. Given the limited sample size and lack of availability of recurrence status for certain patients, we focused on OS. Higher TMB was associated with significantly longer OS (Fig. 7a, HR = 0.85, *p* = 0.036), consistent with previous reports in NSCLC^43^. Conversely, significantly shorter OS was observed in patients with higher CNA burden (Fig. 7b, HR = 1.21, *p* = 0.014). No TCR parameters (T-cell density, richness, and clonality) were associated with OS (Supplementary Fig. 15d–f), however, patients with a more heterogeneous TCR repertoire (lower TCR JI) exhibited significantly shorter OS (Fig. 7c, HR = 0.87, *p* = 0.037).

Interestingly, lower tumor purity, presumably indicative of more immune infiltration, was associated with longer OS (Fig. 7d, HR = 1.30, *p* = 0.001). This finding was further supported by the IHC data on the 66 SCLC patients, where significantly longer OS was observed in patients with higher level of CD3 (Fig. 7e, HR = 0.32, *p* = 0.0167) or positive PD-L1 expression (Fig. 7f, HR = 0.35, *p* = 0.0249). Prolonged DFS was also observed in patients with higher CD3 infiltration (Supplementary Fig. 22a, HR = 0.30, *p* = 0.0059) or positive PD-L1 expression (Supplementary Fig. 22b, HR = 0.54, *p* = 0.1411) in their tumors, suggesting that increased intratumor T-cell infiltration was associated with superior disease control and survival in these SCLC patients.

## Discussion

Although pioneering studies have revealed pivotal molecular features^22–25,38^, the genomic ITH architecture of SCLC has not been defined, primarily due to the lack of adequate tumor specimens for multiregion profiling. Because SCLC is sensitive to initial treatment but nearly all patients experience relapse with refractory disease, it has been speculated that SCLC may have profound mutational ITH, where cancer cells highly resistant to chemotherapy/radiotherapy hide in the treatment-naive SCLC tumors as minor subclones that give rise to relapse^7,44^. Surprisingly, all SCLCs in the current study demonstrated a homogeneous genomic landscape for both mutations and CNAs. Additionally, previous work from our group^45^ and others^38^ has demonstrated striking similarity of the mutational landscape between primary and relapsed SCLC. Taken together, these data indicate that complex genomic ITH and selection of chemo-/radio-resistant minor subclones may not be the main mechanisms underlying therapeutic resistance in SCLC.

Cancer evolution with or without treatment may be shaped by the dynamic interaction between cancer cells and host factors, particularly through immune surveillance^46^. Our study delineates the intratumor TCR repertoire of SCLC and demonstrates an extremely cold TCR repertoire quantitatively (density) and qualitatively (richness and clonality), compared with not only matched normal lung tissues but also compared with NSCLC tumors despite the fact that SCLC and NSCLC tumors had similar TMB and comparable mutational homogeneity. Comparing the immune score and estimated T-cell fraction by deconvolution of transcriptomic data from a previously published larger SCLC cohort (*n* = 81)^23^ to TCGA NSCLC cohorts (*n* = 1,027) also revealed significantly lower immune infiltration in SCLCs than NSCLCs. These findings were further validated orthogonally by IHC using anti-CD3 on 66 SCLC tumors and 68 NSCLC tumors matched for clinicopathological characteristics.

In addition to the cold intratumor TCR repertoire, SCLC tumors also demonstrated an extremely heterogeneous TCR repertoire with only 0.2–14.6% of all T cells identified across all tumor regions within the same tumors. TCR ITH was even more pronounced than that in NSCLC^13^, which may further impair the efficacy of the antitumor immune response. SCLC is among the cancers with high TMB^28^ and our study also demonstrated a homogeneous mutational landscape, both of which have been reported to associate with benefit from ICB^47^. However, compared with NSCLC and other tumor types, fewer SCLC patients benefit from ICB^48^. The cold and heterogeneous TCR repertoire may be one potential reason underlying suboptimal response to immunotherapy.

In subsequent attempts to identify genomic features that may account for the cold TCR repertoire in SCLC, a significantly higher CNA burden was observed in SCLC compared with that in NSCLC. Moreover, the CNA burden was negatively associated with both T-cell quantity (density) and quality (richness and clonality). These results suggest that high CNA burden may be one of the genomic changes underlying the cold TCR repertoire in SCLC. High CNA burden has been reported to correlate with a cold immune microenvironment and inferior benefit from ICB across different cancer types^35,49,50^. The mechanisms underlying the association between high CNA burden and immunosuppression are not well understood. In this study, we observed frequent loss of IFN-γ pathway genes, which has been reported to associate with immune evasion of cancers^39,40^. Importantly, the frequency of IFN-γ pathway gene copy-number loss was associated with the overall CNA burden. One feasible explanation is that dual inactivation of TP53 and RB1 led to global chromosomal instability^51^ (as evidenced by the overall high CNA burden) and higher incidence of losing essential immune genes, including IFN-γ pathway genes, which subsequently empowered SCLCs to evade the host antitumor immune response. Future multiomics studies, including transcriptomic profiling and functional studies, are eventually warranted to determine how exactly CNA impacts the antitumor immune response.

Compared with many malignancies, our understanding of the molecular landscape of SCLC is rudimentary, primarily due to the lack of adequate tumor tissues, as the majority of SCLC patients are not treated with surgical resection. As such, our study was limited by the small sample size, and therefore, these intriguing findings need to be validated by larger cohorts in future studies that may need multi-institutional collaboration. Nevertheless, WES and TCR data from multiregional specimens make these data unique to understand the ITH architecture of SCLC tumors. Additionally, one of the main findings regarding cold T-cell infiltration was validated orthogonally by IHC on a larger cohort of SCLC tumors and NSCLC tumors matched for clinicopathologic features. Another limitation is that it is unclear whether the biology of these resected early-stage SCLC tumors resembles that of advanced-stage SCLCs. On the other hand, we^45^ and Wagner et al^38^ have demonstrated similar genomic landscape between treatment-naive resected SCLC tumors and recurrent disease, suggesting that some of the findings in the current study may be applicable to advanced SCLCs. Finally, the resected tumors only offer one molecular snapshot during evolution of these SCLC tumors and the dynamic interaction between cancer cells and immune features before the surgical resection is still unknown. For example, although the low T-cell infiltration, low immune score, and low PD-L1 expression suggested an overall cold immune response rather than the high level of adaptive immune evasion in these SCLC tumors, the high prevalence of HLA LOH suggested that active adaptive immune suppression may have taken place at some point during early evolution of these SCLC tumors.

In summary, we demonstrate that despite a homogeneous genomic landscape, SCLC exhibits a cold and heterogeneous T-cell infiltration that could potentially lead to ineffective antitumor immune surveillance. From a therapeutic standpoint, these findings demand further investigation into approaches capable of overcoming the cold and heterogeneous intratumor T cell repertoire to improve the efficacy of immunotherapy for SCLC patients. Enhancement of T-cell trafficking into SCLC tumors as well as within tumors may have the potential to promote T-cell infiltration and make the immune infiltrate more consistent, therefore generating more effective antitumor responses. T-cell trafficking can be impaired by tumor-vascular structure^52^, aberrant tumor extracellular-matrix architecture^53^, presence of myeloid-derived suppressor cells (MDSCs)^54^, hypoxia^55^, lack of T-cell recruitment-associated chemokines such as CCL5 and CXCL9^56^, as well as other mechanisms. Targeting these pathways to reprogram T-cell motility may provide opportunities to improve the efficacy of immunotherapy in tumors with a cold and/or heterogeneous immune infiltrate such as SCLC.

## Methods

### Patients

A total of 67 patients with LS-SCLC (11 females and 56 males with median age 64 (range: 38–82)) who underwent surgical resection at Zhejiang Cancer Hospital, Hangzhou, China from 2008 to 2020, were enrolled. All the enrolled patients underwent upfront surgery without preoperative chemotherapy or radiation therapy and no patients received immunotherapy prior to or post surgery. As a control group, 68 patients with localized NSCLC (18 females and 50 males with median age 64 (range: 36–79)), who underwent upfront surgery without preoperative chemotherapy or radiation therapy also at Zhejiang Cancer Hospital, Hangzhou, China, were included. The clinical information of all 135 patients is included in Supplementary Data 1. The SCLC group and NSCLC group were matched for clinical characteristics, including gender, age, smoking status and tumor size (Table 1). Written informed consent was obtained from all patients involved. The study was approved by the Institutional Review Boards (IRB) at MD Anderson Cancer Center and Zhejiang Cancer Hospital. This study is compliant with the “Guidance of the Ministry of Science and Technology (MOST) for the Review and Approval of Human Genetic Resources”, which requires formal approval for the export of human genetic material or data from China.

### Sample processing and DNA extraction

Hematoxylin and eosin (H&E) slides from each tumor were reviewed by experienced lung-cancer pathologists to confirm the diagnosis, assess necrosis, tumor purity, and cell viability. Manual macrodissection was conducted to enrich malignant cells. A H&E slide from each tumor region was first reviewed by experienced lung-cancer pathologists to assess the percentage of tumor versus adjacent normal tissues. Three regions from each tumor FFPE block, were collected by a “grid” approach representing the spatial heterogeneity of the primary tumors. Only tumor regions with sufficient tumor cells (estimated 50,000 cancer cells) that could yield a minimum of 150 ng of DNA were selected for DNA exaction and sequencing (Supplementary data 3, Supplementary Fig. 2). DNA was extracted using the AllPrep^®^ DNA/RNA FFPE Kit (Qiagen, Hilden, Germany) from 50 spatially separated tumor regions from 19 LS-SCLC tumors with adequate tissues available (three regions per tumor from 13 patients, two regions per tumor from five patients, and one tumor piece from one patient) and paired matched adjacent normal lung (≥2 cm from tumor margin, morphologically negative for malignant cells assessed by two lung cancer pathologists independently)^57^.

### WES

WES was performed using the Illumina protocol at MD Anderson Cancer Center. DNA was extracted using the QIAamp DNA FFPE Tissue Kit (QIAGEN) and the resulting genomic DNA was sheared into 300–400-bp segments and subjected to library preparation for WES using KAPA library prep (Kapa Biosystems) with the Agilent SureSelect Human All Exon V4 kit according to the manufacturer’s instructions.^58^ In all, 76-nt paired-end multiplex sequencing of DNA samples was performed on the Illumina HiSeq 2500 sequencing platform. The average sequencing depth was 180x for tumor DNA (ranging from 64x to 224x), 161x for germline DNA (ranging from 96x to 194x).

### Quality control for sequencing data from FFPE samples

As all specimens were formalin-fixed paraffin-embedded (FFPE) samples, which are known to be associated with artifacts from NGS, rigorous quality control was applied before further analyses^57^. FFPE artifacts usually present as nonrecurrent, low log-odds (LOD) score, and low variant allele frequency (VAF) (usually < 10%), predominantly C > T | G > A “transitions”. On the other hand, smoking-induced mutations are predominately C > A | G > T. Therefore, in addition to sequencing depth, VAF, and minimal counts of alternative reads, a minimal LOD threshold of 18 (the default is 6.3 for somatic mutation calls) based on our data was applied to filter out FFPE artifacts. As shown in Supplementary Fig. 23, “mutations” with low LOD scores exhibited a high proportion of C > T | G > A transitions, while mutations with high LOD scores showed consistent proportion of C > A | G > T transversions, suggesting that “mutations” with low LOD scores were likely “contaminated” by FFPE artifacts. We then assessed the quality of mutation calls after our stringent filtering. As shown in Supplementary Fig. 9, the predominant single nucleotide variants (SNVs) included in the current study were predominantly C > A | G > T rather than C > T | G > A. Taken together, these data suggest that FFPE artifacts were controlled for the current study.

### Mutation calling

The BWA aligner (bwa-0.7.5a) was applied to map the raw reads to the human hg19 reference genome (UCSC genome browser: genome.ucsc.edu). The Picard (v1.112, http://broadinstitute.github.io/picard/) “MarkDuplicates” module was applied to mark the duplicate reads. Then the “IndelRealigner” and “BaseRecalibrator” modules of the Genome Analysis Toolkit were applied to perform indel realignment and base-quality recalibration. Mutect (v1.1.4)^59^ was applied to identify SNVs and small insertions/deletions. To ensure high-quality mutation calls, the following filtering criteria were applied: (1) sequencing depth ≥20× in tumor DNA and ≥10× in germline DNA; (2) VAF ≥0.02 in tumor DNA and <0.01 in germline DNA; (3) the total number of reads supporting the variant calls is ≥4; (4) variant frequency is <0.01 in ESP6500, 1000-genome, and EXAC databases; and (5) LOD score >18 (MuTect default is 6.3). We kept the mutations that passed all filtering criteria except LOD score <18, only if the identical mutations were present with LOD score > =18 in other regions within the same tumors. Cancer gene mutations were defined as identical oncogene mutations previously reported, stop gains and frameshift of tumor suppressor genes, and other nonsynonymous mutations with Combined Annotation Dependent Depletion (CADD) score >20^60^.

### Clonal analysis

To delineate the clonal architecture of the 19 SCLC tumors, we merged the bam files from different regions within the same tumors and calculated the percent of clonal and subclonal mutations. To ensure high-quality mutation calls at tumor level, the following filtering criteria were applied: (1) sequencing depth ≥20× in tumor DNA and ≥10× in germline DNA; (2) variant-allele frequency (VAF) ≥0.02 in tumor DNA and <0.01 in germline DNA; (3) the total number of reads supporting the variant calls is ≥4; (4) variant frequency is <0.01 in ESP6500, 1000-genome, and EXAC databases; and (5) LOD score >30 (MuTect default is 6.3) based on our data (Supplementary Fig. 24). We rescued those mutations if they were identified at region level. Tumor contents and major/minor copy number changes were then estimated by Sequenza (v2.1.2)^31^. Pyclone-VI^61^ was applied to estimate the clonal status of each mutation. In brief, PyClone implements a Dirichlet process clustering model that simultaneously estimates the distribution of the cellular prevalence for each mutation. Copy numbers of somatic mutations were inferred by integrating integer copy numbers determined by Sequenza (v2.1.2) on single-sample basis. The outputs were cellular-prevalence value distributions per SNV estimated from Markov-chain Monte Carlo (MCMC) sampling. The median value of the MCMC sampling-derived distribution was used as a representative cellular prevalence for each mutation. Those mutations in the cluster with the highest cellular prevalence were classified as “clonal”, otherwise, “subclonal”^62^.

### Phylogenetic analysis

Mutation profiles were converted into binary format with 1 being mutated and 0 otherwise. Ancestors were germline DNA assuming with no mutations. Multistate discrete-character Wagner parsimony method in PHYLIP (Phylogeny Inference Package) (version 3.695) was used to generate phylogenic tree^63^.

### Mutational signature analysis

The R package “DeconstructSigs” (version 1.8.0)^64^ was applied to estimate the proportions of 30 COSMIC mutational signatures (http://cancer.sanger.ac.uk/cosmic/signatures).

### Somatic copy number analysis

ExomeCNV (version 1.4)^65^ was applied to infer CNA by estimating the tumor/normal read-count log2 ratio of the capture region followed by segmentation. CNTools package (version 1.49.0) was used to segment DNA copy number profiles at the gene level. Those genes with mean-segment log2 ratio >0.6 were defined as copy number gain and < −0.6 was defined as copy number loss. CNA burden was quantified using a gene-based CNA analysis algorithm^26^ for exome-sequencing data that allows the comparison of the CNA between different samples. To define the copy-number gains of oncogenes identified by ExomeCNV, the copy number output from Sequenza was taken into consideration. Only oncogenes with copy number values higher than the overall ploidy of the sample in Sequenza output were defined as having copy-number gains.

### Neoantigen prediction

WES data were reviewed for nonsynonymous exonic mutations. The binding affinity with patient-restricted MHC class I molecules of all possible 9- and 10-mer peptides was evaluated with the NetMHC3.4 algorithm based on patient HLA-A, HLA-B, and HLA-C alleles^66–68^. HLA allele was predicted by POLYSOLVER (version 1.2.0)^41^. Candidate peptides were considered HLA binders when IC50 < 500 nM.

### TCR-β sequencing and comparison parameters

Immunosequencing of the CDR3 regions of human TCR-β chains was performed using the protocol of ImmunoSeq (Adaptive Biotechnologies, hsTCRβ Kit)^69–71^. Two sets of PCRs were performed on DNA extracted from the tissues collected. The initial PCR used a mix of multiplexed V- and J-gene primers that amplify all possible recombined receptor sequences from the DNA sample, and then a second PCR designing to add unique DNA barcodes to each PCR product was followed. After that, samples were pooled together with a negative and a positive control. The pools were then sequenced on an Illumina MiSeq platform using a 150-cycle paired-end protocol and sequence-ready primers. After sequencing, the raw data were transferred to Adaptive Biotechnologies and processed into a report, including those that passed quality-check samples and a normalized and annotated TCR-β-profile repertoire accordingly.

T-cell density was calculated by normalizing TCR-β template counts to the total amount of DNA usable for TCR sequencing, where the amount of usable DNA was determined by PCR amplification and sequencing of housekeeping genes expected to be present in all nucleated cells. T-cell richness is a metric of T-cell diversity, and it is calculated on the T-cell unique rearrangements. T-cell clonality is a metric of T-cell proliferation and reactivity, and it is defined as 1-Peilou’s evenness. Clonality ranges from 0 to 1: values approaching 0 indicate a very even distribution of the frequency of different clones (polyclonal), whereas values approaching 1 indicate a distinct asymmetric distribution in which a few activated clones are present at high frequencies (monoclonal). Statistical analysis was performed in R version 3.2 in ImmunoSEQ ANALYZER (ANALYSES 3.0). The immunoSEQ Assay is for research use only and not for use in diagnostic procedures. TCR Jaccard index (JI) is conceptually a percentage of how many objects of two sets have in common out of how many objects they have in total. JI was calculated by the number of rearrangements shared/sum of the total number of rearrangements between any two specimens. Morisita index (MOI) is a measure of the similarity in the T-cell repertoire between samples ranging from 0 to 1, taking into account the specific rearrangements and their respective frequencies, with an MOI of 1 being an identical T-cell repertoire.

### Human-leukocyte antigen loss of heterozygosity analysis

Loss of heterogeneity (LOH) of human-leukocyte antigen (HLA) was assessed^41^. Briefly, class-I HLA alleles for each HLA gene were inferred from germline DNA by POLYSOLVER (version 1.2.0) using a two-step Bayesian classification approach, which takes into account the base qualities of aligned reads, observed insert sizes, as well as the ethnicity-dependent prior probabilities of each allele. Tumor purity and ploidy were then estimated using Sequenza (v2.1.2)^31^, and LOHHLA (Loss of Heterozygosity in Human Leukocyte Antigen) algorithm was applied to detect allele-specific HLA loss in each tumor sample. Briefly, logR and BAF across each HLA gene locus was obtained by binning the coverage at mismatch positions between homologous HLA alleles, and HLA haplotype-specific copy numbers were then calculated based on logR and BAF values from the corresponding bin adjusted by tumor purity and ploidy. The median value of binned allelic copy number was used to determine LOH, where a copy number of <0.5 indicated allele loss and LOH was determined if *p* < 0.01.

### Copy number LOH analysis

For each sample, we calculated the fraction of copy-number losses for those genes associated with predicted neoantigens versus other mutated genes not associated with predicted neoantigens, respectively. The segment copy-number file (predicted by Sequenza (v2.1.2)^31^) was used to evaluate a gene showing evidence of copy number neutral LOH (CNN–LOH) or copy number loss LOH (CNL–LOH). Those candidate genes within the chromosomal segments with the count of A allele equaling to 2 and B allele equaling to 0 were considered as CNN–LOH, while counts of A allele equaling to 1 and B allele equaling to 0 were considered as CNL–LOH^72^. For those mutated genes not associated with predicted neoantigens, the fraction was calculated by the total number of mutated genes with CNN–LOH/CNL–LOH divided by the total number of mutated genes. For those predicted neoantigen-associated genes, the fraction was calculated by the total number of predicted neoantigen-associated genes with CNN–LOH/CNL–LOH divided by the total number of genes associated with predicted neoantigens.

### Immunohistochemistry

Immunohistochemistry (IHC) of CD3 and PD-L1 was performed on 66 LS-SCLC tumors (including the same 18/19 tumors that underwent multiregion WES and TCR sequencing) and 68 localized NSCLC tumors with matched clinical characteristics including gender, age, smoking status and tumor size (Supplementary Data 1 and Table 1). Representative IHC figures are shown in Supplementary Fig. 14. Serial sections with a thickness of 4 μm from FFPE samples were cut onto glass slides, followed by IHC staining. PD-L1 IHC testing was performed using the PD-L1 clone 22C3 pharmDx kit and Dako Automated Link 48 platform (Cat No. SK006, Agilent Technologies/Dako, Carpinteria, CA, USA. This antibody was provided by Merck & Co., Inc.). Slides stained with CD3 were labeled by a mouse anti-CD3 monoclonal antibody (BOND™ Ready-to-Use Primary Antibody CD3 (LN10) (no dilution), Cat No. PA0553, Leica Biosystems, Newcastle Upon Tyne, UK; https://shop.leicabiosystems.com/zh-cn/ihc-ish/ihc-primary-antibodies/pid-cd3) at a working solution and incubated on an autostainer (Leica BOND-III, Leica Biosystems, Newcastle Upon Tyne, UK). Two independent observers examined the stained slides in a blinded fashion. The PD-L1 tumor-proportion score (TPS) values were determined by the formula (PD-L1-positive tumor cells/total tumor cells*100%) in the slides. The PD-L1-positive tumor cells were defined by clear membrane staining tumor cells with or without plasma staining at any extent. For each slide, CD3-positive tumor-infiltrating lymphocytes (TILs) were indicated by the values (CD3 + TILs/tumor cells) in each of 10 typical high-power phases (20×).

### Analysis of published data

RNA sequencing data from 81 SCLCs^23^ and 1027 NSCLCs from TCGA^30,32^ were downloaded. The estimated T-cell fraction was calculated by MCPcounter (version 1.0.0)^73^. The immune score was calculated with the R package “ESTIMATE” (https://sourceforge.net/projects/estimateproject/), which infers the infiltration of immune cells by 141 gene expression signatures^74^. Copy number comparison of this study makes use of data generated by The TRAcking Non-small Cell Lung Cancer Evolution through Therapy (Rx) (TRACERx) Consortium and provided by the UCL Cancer Institute and The Francis Crick Institute. The TRACERx study is sponsored by University College London, funded by Cancer Research UK, and coordinated through the Cancer Research UK and UCL Cancer Trials Center.

### Statistical analysis

Graphs were generated with GraphPad Prism 8.0 (La Jolla, CA). Pearson’s correlations were calculated to assess the association between two continuous variables. Wilcoxon signed-rank test was applied to compare two paired groups. Mann–Whitney test was used to compare differences between two independent groups. Kruskal–Wallis H test was applied to compare categorical variables with more than two levels. Chi-squared test was used to compare categorical variables in two groups. Log-rank test was used for survival analysis.

### Reporting summary

Further information on research design is available in the Nature Research Reporting Summary linked to this article.

## Supplementary information

Supplementary information

Description of Additional Supplementary Files

Supplementary Dataset 1

Supplementary Dataset 2

Supplementary Dataset 3

Reporting Summary

## Data Availability

The WES data in this study have been deposited in the European Bioinformatics Institute European Genome–phenome Archive (EGA) (accession number: EGAS00001005087) through controlled access. TCR sequencing data in this study are available through the immuneACCESS platform (10.21417/MC2021NC; [https://clients.adaptivebiotech.com/pub/chen-2021-nc]). To protect patient privacy, researchers interested in the WES data need to apply via data-access committee (DAC) by contacting Dr. Jianjun Zhang at Jzhang20@mdanderson.org, which will grant all reasonable requests. TCR sequencing data have been shared as public data. The WES data of PROSPECT cohort used in this study are available in the EGA database under accession code EGAS00001004026. TCR sequencing data of PROSPECT cohort are available through the immuneACCESS platform (10.21417/AR2019NC [https://clients.adaptivebiotech.com/pub/reuben-2019-natcomms]). Sequencing data of George cohort used in this study are available in the EGA database under the accession code EGAS00001000925. Sequencing data of TRACERx cohort used in this study are available in the EGA database under the accession code EGAS00001002247. Sequencing data of TCGA used in this study are available in the Genomic Data Commons (GDC) and the Data Coordinating Center (DCC) for public access (https://gdc.cancer.gov/ and https://www.cancer.gov/about-nci/organization/ccg/research/structural-genomics/tcga). All other data may be found within the Article file, Supplementary information, or Source Data file. Source data are provided with this paper.

## Source data

Source Data
