# Supplementary material for: Cold and heterogeneous T cell repertoire is associated with copy number aberrations and loss of immune genes in small-cell lung cancer
Source: Nat Commun. 2021 Nov 17;12:6655. doi: 10.1038/s41467-021-26821-8 (PMC8599854; doi:10.1038/s41467-021-26821-8)
Supplement: Supplementary file 1 — Supplementary information [file 41467_2021_26821_MOESM1_ESM.pdf]

Supplementary information

Cold and heterogeneous T cell repertoire is associated with copy number aberrations and loss of immune genes in small-cell lung cancer

Chen et al.

Supplementary Figure 1

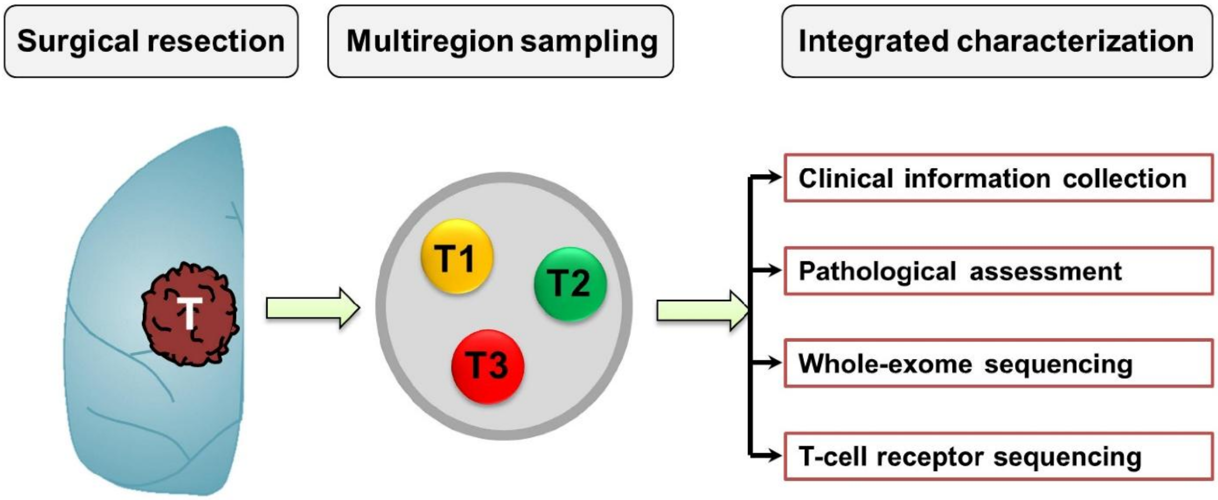

Supplementary Fig. 1 Study scheme of genomic and T cell receptor (TCR) intratumor heterogeneity (ITH) of small-cell lung cancer (SCLC) by multi-region sequencing.

Supplementary Figure 2

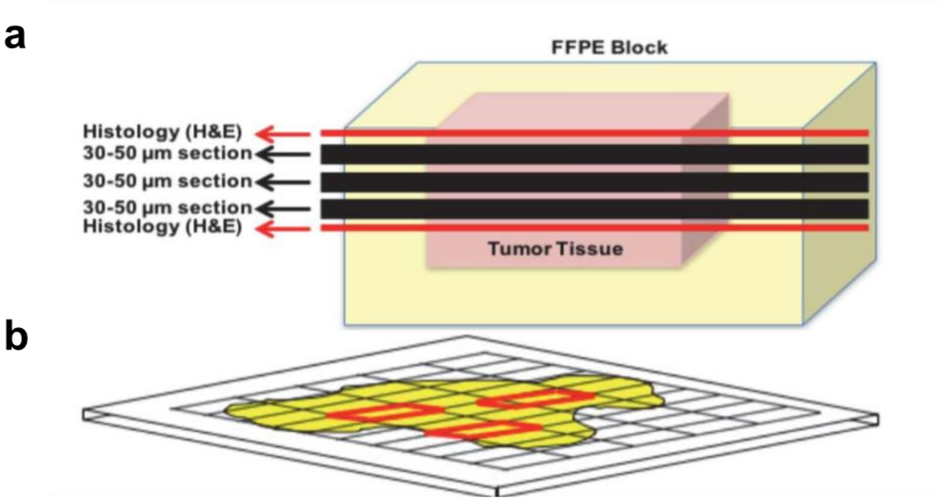

Supplementary Fig. 2 Schema of tissue "grid" methodology for multi-region sampling. (a) Tissue is consecutively sectioned for histology evaluation (H&E), 30-50

µm section, followed by 5 µm histology sections for H&E assessment. **(b)** A 50µm thick section from a large lesion like surgically resected lung adenocarcinoma. A grid is superimposed between H&E and thick sections to identify and select multiple spatially separated 3 x 3 mm regions (red squares) to be dissected for DNA extraction using Biobank Card system.

Supplementary Figure 3

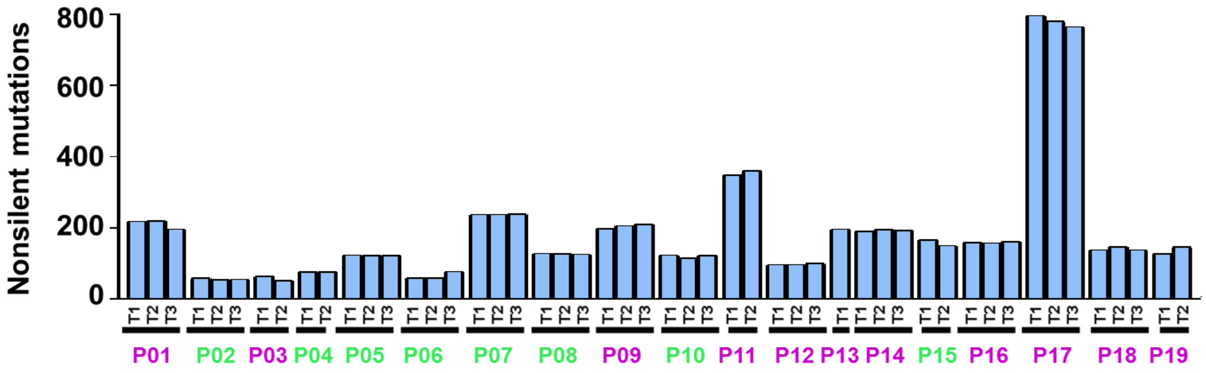

Supplementary Fig. 3 Non-silent mutations in 50 small-cell lung cancer (SCLC) specimens. Total number of non-silent mutations in 50 SCLC tumors. Patient ID: purple = alive; green = deceased. Source data are provided as a Source Data file.

Supplementary Figure 4

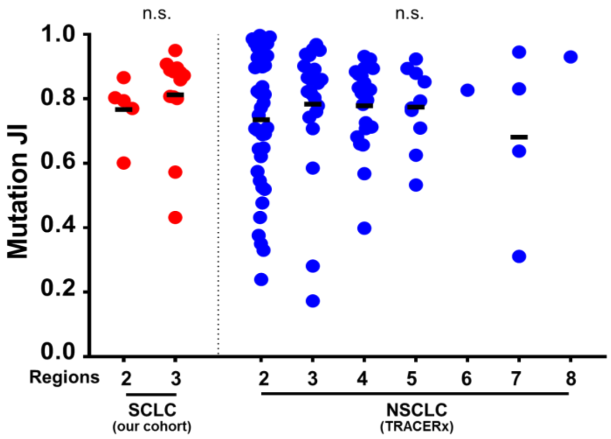

Supplementary Fig. 4 The impact of number of tumor regions on assessing genomic heterogeneity using Jaccard index (JI) in small-cell lung cancer (SCLC) and non-small cell lung cancer (NSCLC) tumors. Genomic intratumor heterogeneity (ITH) using the average Jaccard index (JI), a metric representing the proportion of shared mutations between two samples in 18 SCLC (blue) *versus* 100 NSCLC tumors (red) from TRACERx with multi-region genomic data. The x-axis represents the numbers of spatially separated tumor regions in each tumor. The difference of region number of SCLC was evaluated using two-sided Mann-Whitney test. The difference of region number of NSCLC was evaluated using two-sided Kruskal-Wallis H test. Source data are provided as a Source Data file.

Supplementary Figure 5

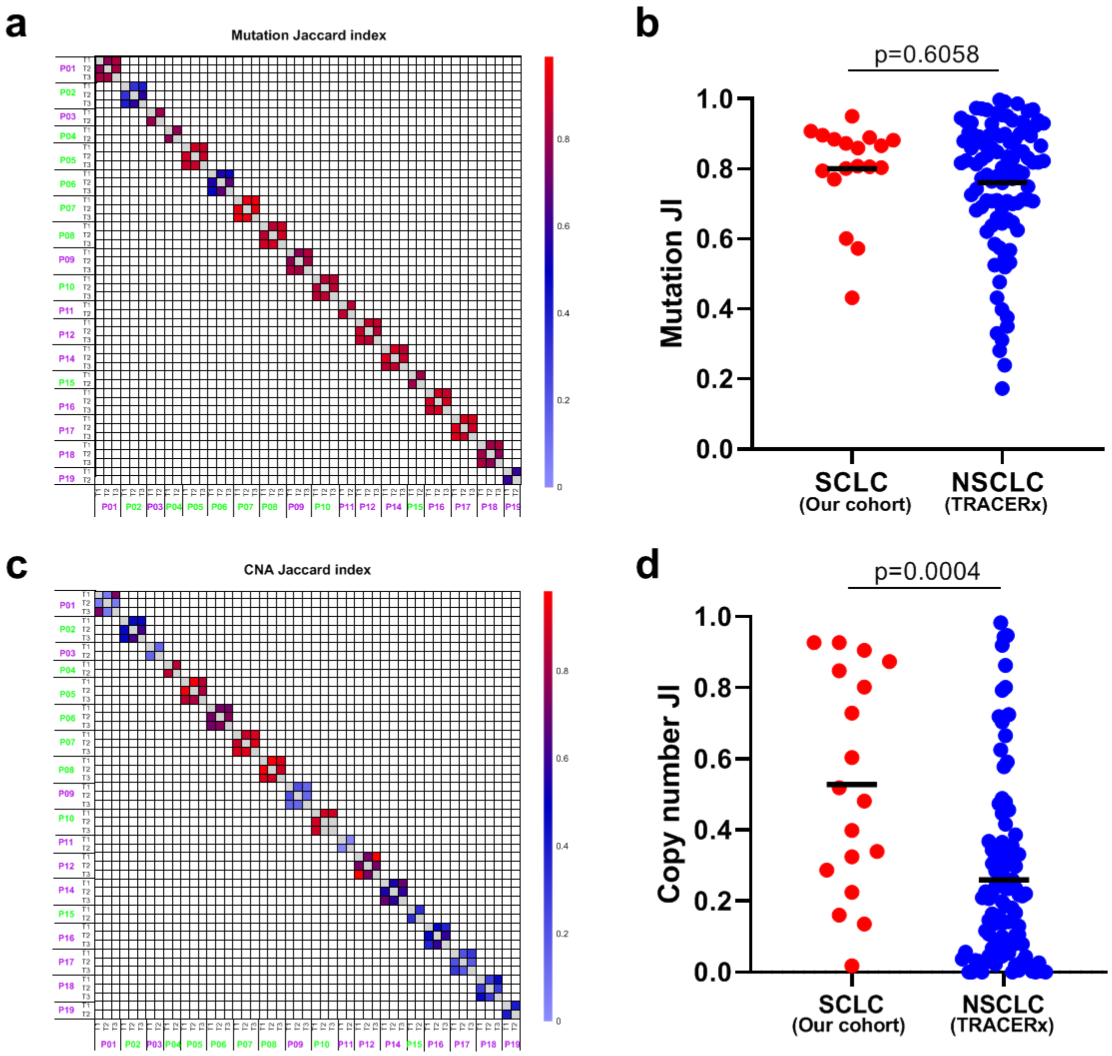

Supplementary Fig. 5 Genomic intratumor heterogeneity (ITH) in small-cell lung cancer (SCLC) and comparison with non-small cell lung cancer (NSCLC) tumors from TRACERx cohort. (a) Quantification of mutation ITH by Jaccard index (JI), a metric representing the proportion of shared somatic mutations between two samples. **(b)** Average mutation JI, a metric representing the proportion of shared mutations between two samples in 18 SCLC (blue) *versus* 100 NSCLC tumors (red) from TRACERx with multi-region genomic data. **(c)** Quantification of copy number ITH by Jaccard index (JI), a metric representing the proportion of shared copy number aberrations (CNA) between two samples. **(d)** Average copy number JI, a metric

representing the proportion of shared CNAs between two samples in 18 SCLC (red) *versus* 100 NSCLC tumors (blue) from TRACERx with multi-region genomic data. The difference of JI between SCLC and NSCLC was evaluated using two-sided Mann-Whitney test. Source data are provided as a Source Data file.

Supplementary Figure 6

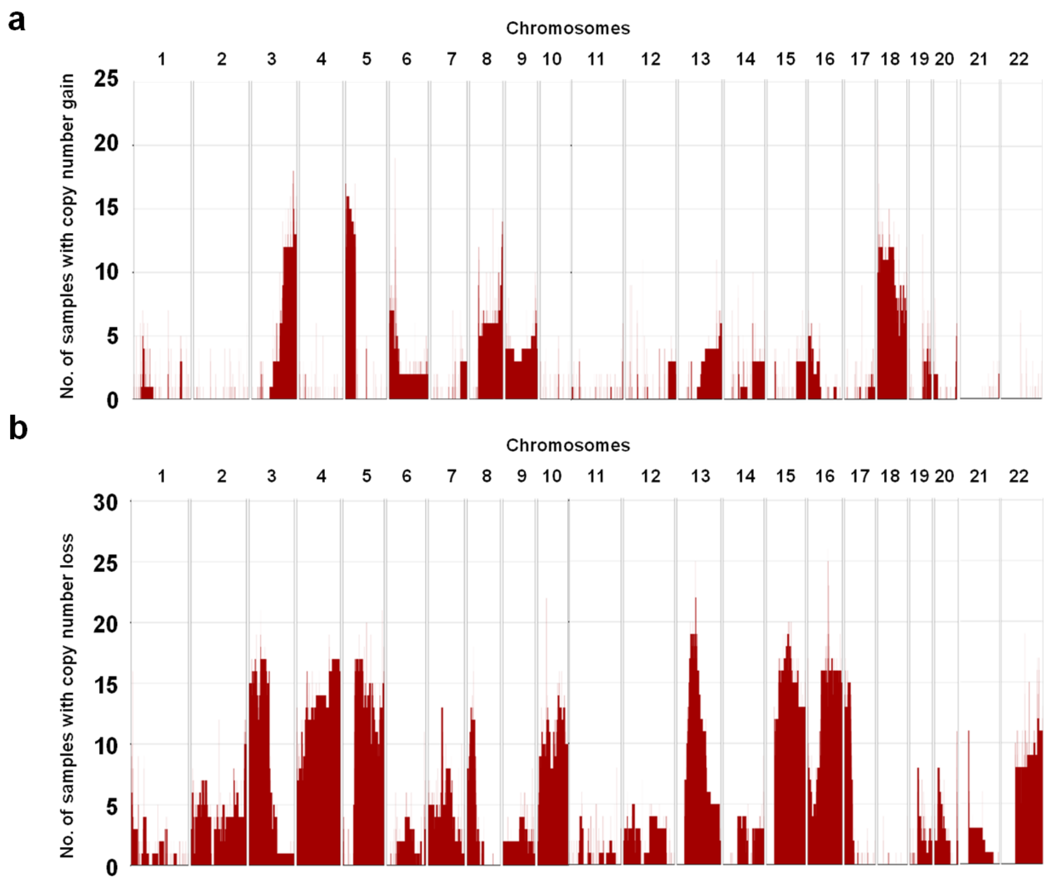

Supplementary Fig. 6 Copy number alteration (CNA) profiles in 50 SCLC samples. The distribution of copy number **a)** gain and **b)** loss at different chromosomal locations across the 50 SCLC samples from 19 patients. Y-axis represents number of samples. Source data are provided as a Source Data file.

Supplementary Figure 7

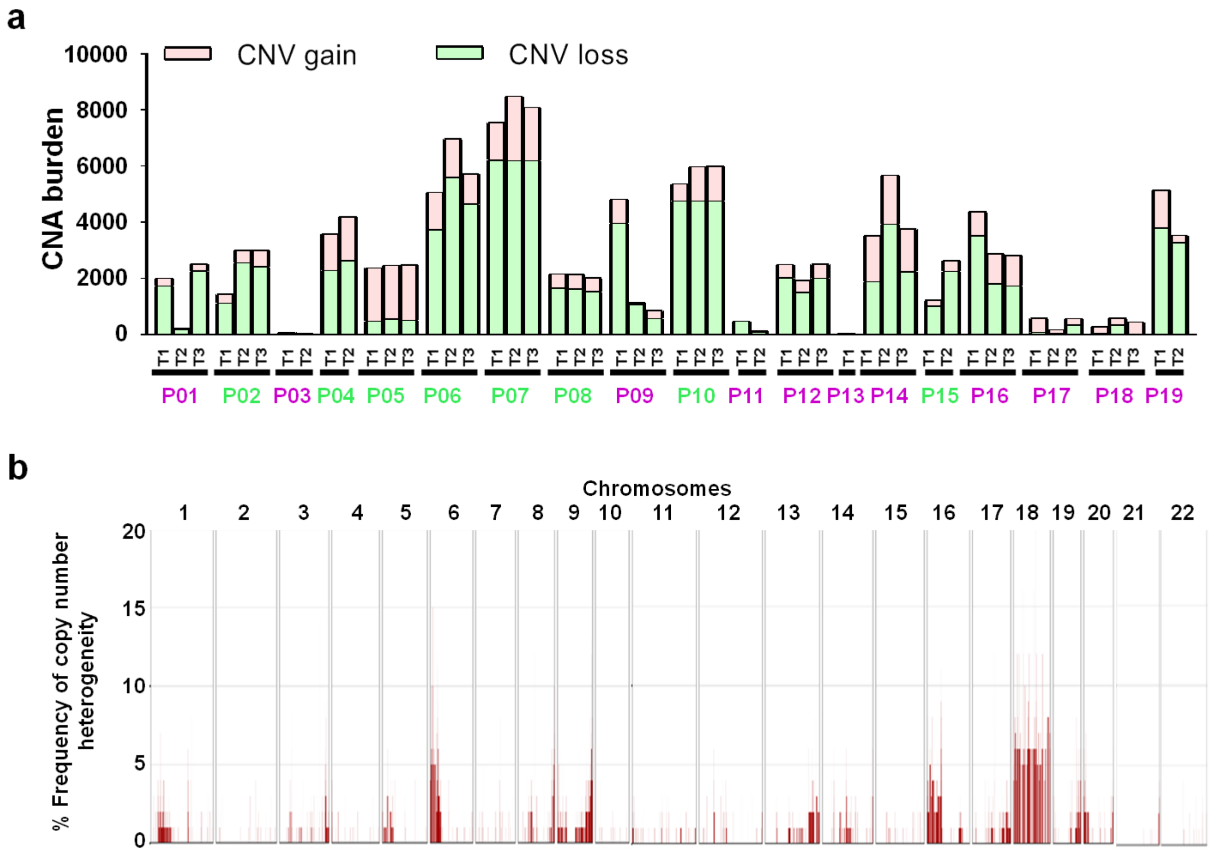

Supplementary Fig. 7 Copy number aberration (CNA) burden in 50 small-cell lung cancer (SCLC) specimens. (a) Copy number gain burden (number of genes in the chromosomal segments with tumor/normal log2 ratio ≥ 0.6, peach) and loss burden (number of genes in the chromosomal segments with tumor/normal log2 ratio ≤ -0.6, green) in 50 SCLC tumors. Patient ID: purple = alive; green = deceased. **(b)** Frequency of copy number alteration (CNA) heterogeneity in SCLCs by different chromosomal locations. The CNA heterogeneity was defined as inconsistent CNA status present in different tumor regions within the same tumors. Frequency of CNA heterogeneity represents the fraction of CNAs that were not identified in all 3 regions within a given tumor. Source data are provided as a Source Data file.

Supplementary Figure 8

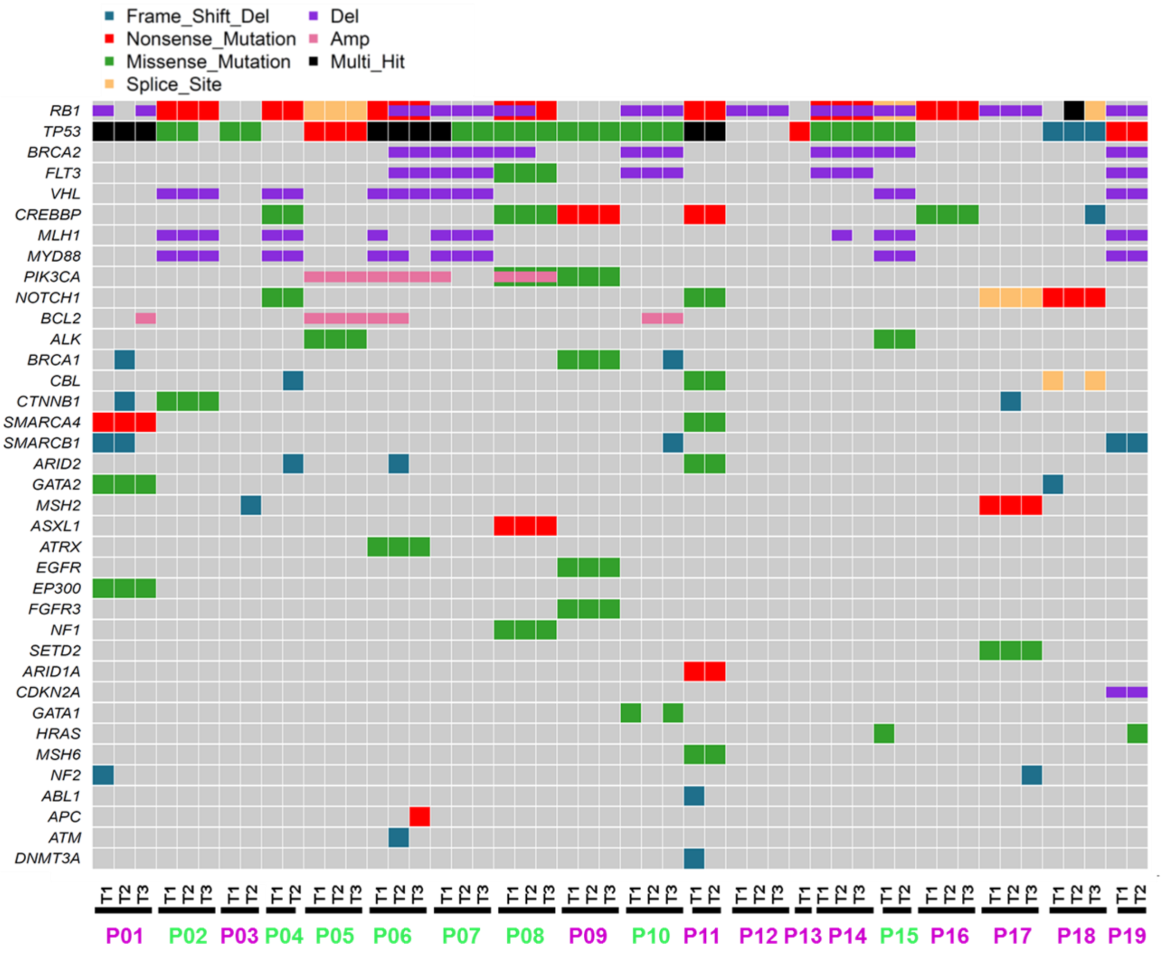

Supplementary Fig. 8 Cancer gene alterations in 50 small-cell lung cancer (SCLC) specimens from 19 patients. Cancer gene mutations were defined as identical to mutations previously reported in oncogene, stop-gain or frameshift mutations in tumor suppressor genes and other non-silent mutations with Combined Annotation Dependent Depletion (CADD) score>20. Copy number loss of tumor suppressors was defined as the mean segment log2 ratio < -0.6. Copy number gain of oncogenes was defined as the mean segment log2 ratio >0.6 and log2 ratio of segment/log2 ratio of chromosomal arm > 1.3. Multi-hit was defined as presence of different unique alterations of the same cancer genes. Purple patient IDs = alive; Green patient IDs = deceased. Source data are provided as a Source Data file.

Supplementary Figure 9

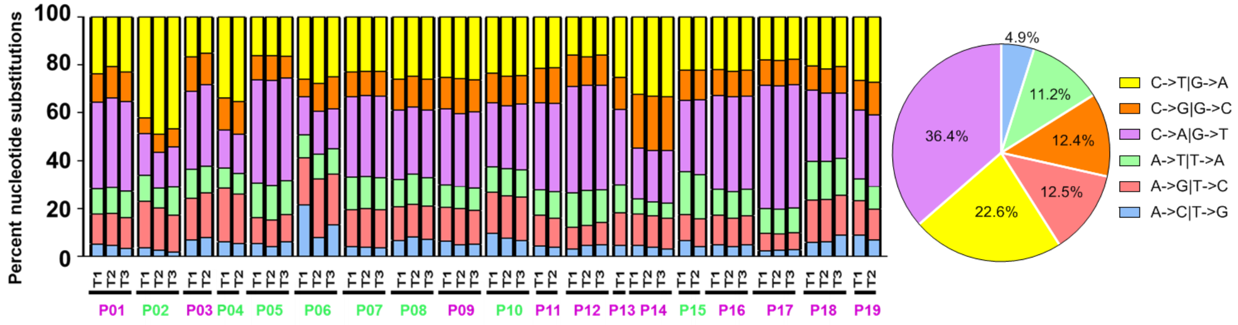

Supplementary Fig. 9 Mutational spectrum and the distribution of nucleotide substitutes in 50 small-cell lung cancer (SCLC) specimens. Purple patient IDs = alive; Green patient IDs = deceased.

Supplementary Figure 10

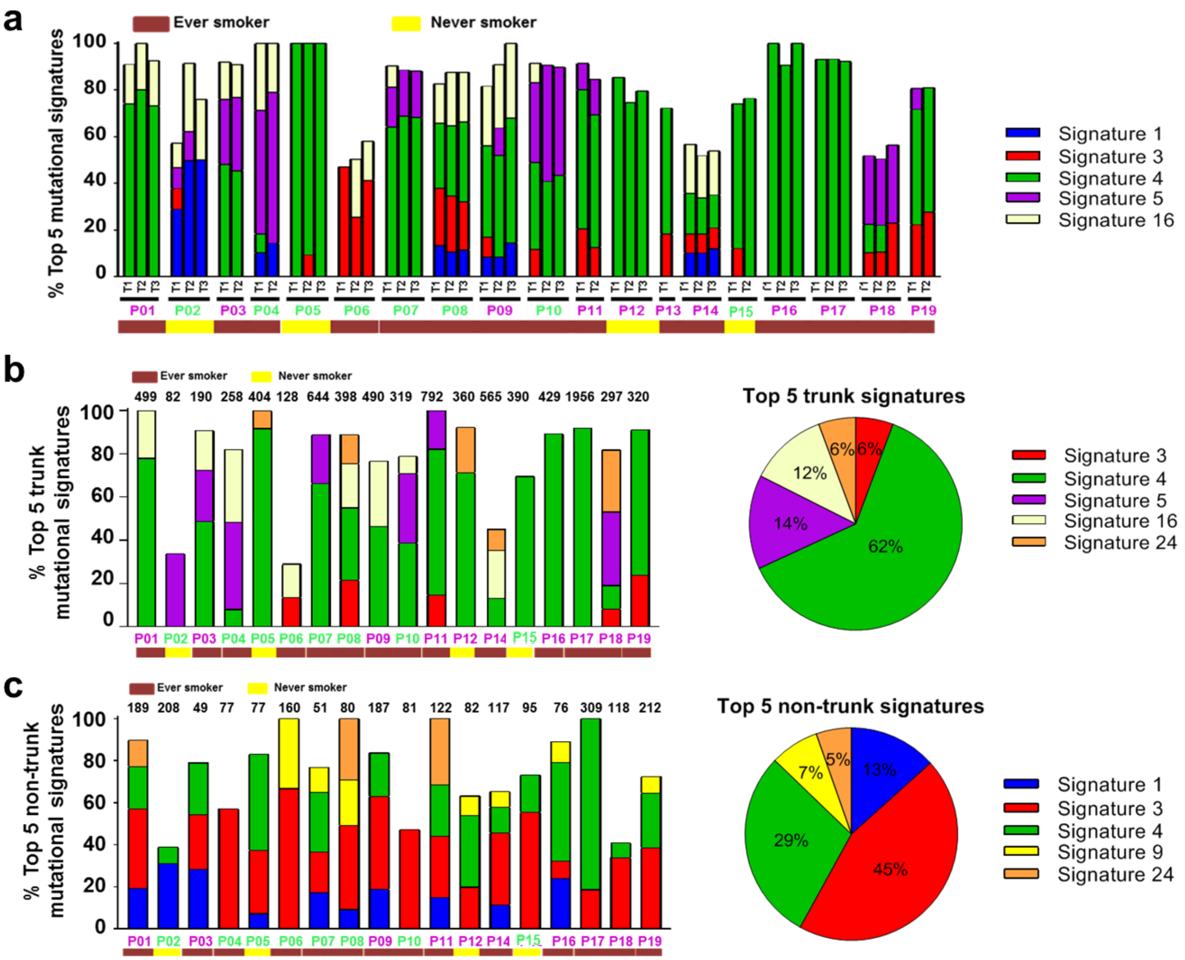

Supplementary Fig. 10 The mutational processes during early versus late mutagenesis of small-cell lung cancer (SCLC). (a) The top 5 COSMIC mutational signatures in 50 SCLC specimens. **(b)** The top 5 COSMIC mutational signatures associated with trunk mutations. Bar chart on the left: top signatures associated with trunk mutations in each patient. Pie chart on the right: the average of contribution of each signature across the 18 patients. **(c)** The top 5 COSMIC mutational signatures associated with non-trunk mutations. Bar chart on the left: top signatures associated with non-trunk mutations in each patient. Pie chart on the right: the average of contribution of top signatures across the 18 patients. Purple patient IDs = alive; Green patient IDs = deceased. Source data are provided as a Source Data file.

Supplementary Figure 11

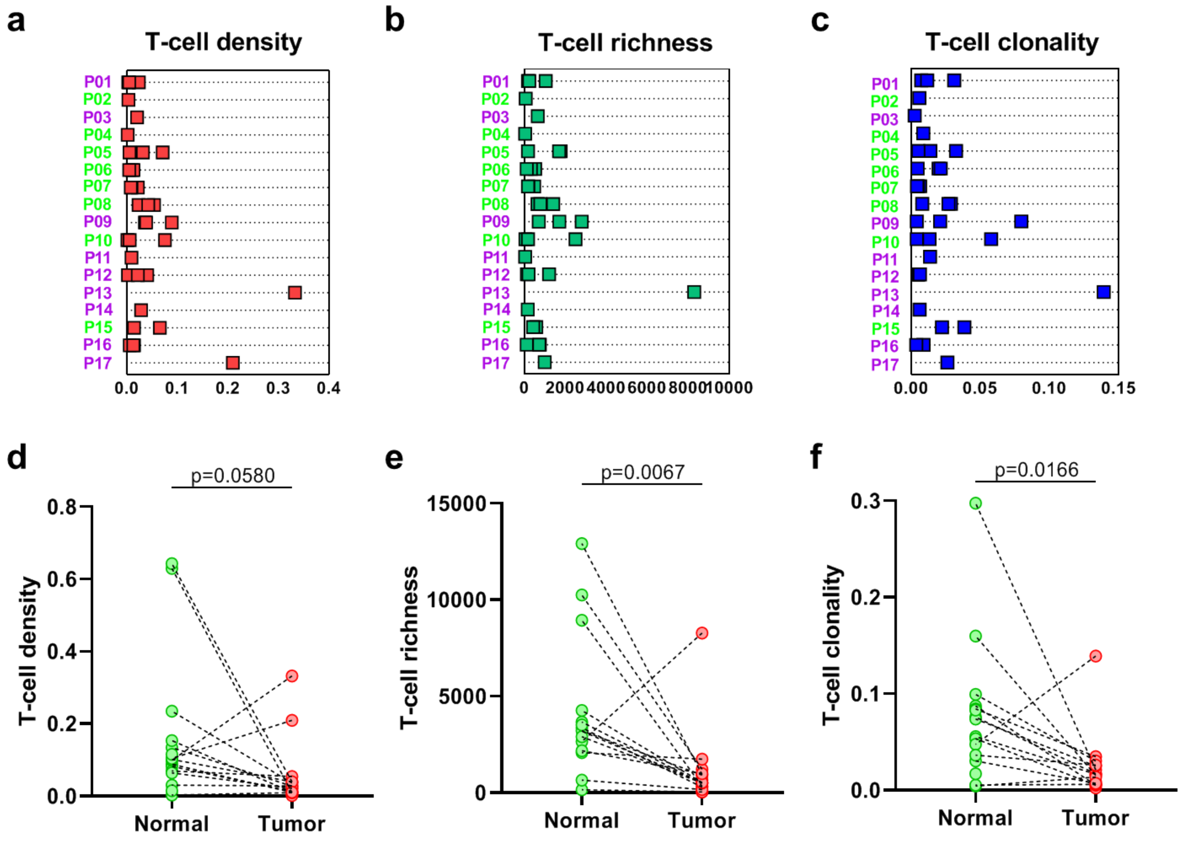

Supplementary Fig. 11 T cell receptor (TCR) repertoire landscape of small-cell lung cancer (SCLC). (a) T-cell density (red) - an estimate of the proportion of T cells in a tumor specimen, **(b)** T-cell richness (green) - a measure of T-cell diversity in a tumor specimen, and **(c)** T-cell clonality (blue) - a metric indicating T-cell expansion and reactivity a tumor specimen in 36 samples from 17 SCLC patients with TCR data available. Purple patient IDs = patients who were alive; Green patient IDs = patients who were deceased; median follow up time: 53 months. T-cell **(d)** density, **(e)** richness and **(f)** clonality in tumors (red) *versus* normal tissues (green) in 14 SCLC patients with both TCR data of tumor and normal tissues available. Two-sided Wilcoxon signed-rank test was applied. Source data are provided as a Source Data file.

Supplementary Figure 12

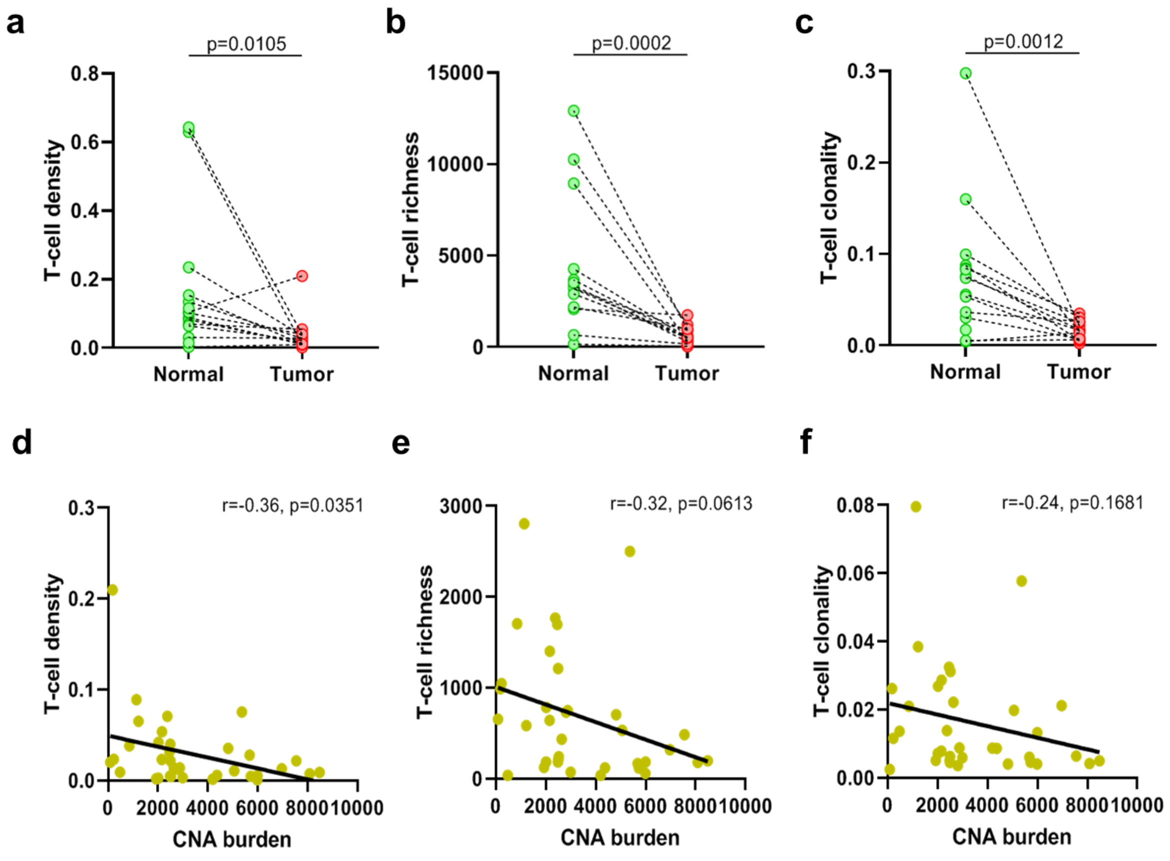

Supplementary Fig. 12 Comparison of T cell receptor (TCR) metrics between small-cell lung cancer (SCLC) tumors and normal lung tissues and correlations between copy number aberration (CNA) burden with TCR metrics in SCLCs without the outlier P13. T-cell **(a)** density, **(b)** richness and **(c)** clonality in tumors (red) *versus* normal tissues (green) with P13 excluded in 35 samples from 16 SCLC patients with TCR data available. Two-sided Wilcoxon signed-rank test was applied. Correlations of CNA burden with **(d)** T-cell density, **(e)** T-cell richness and **(f)** T-cell clonality in 35 SCLC samples with P13 excluded. Two-sided Pearson's correlations were calculated to assess associations. Source data are provided as a Source Data file.

Supplementary Figure 13

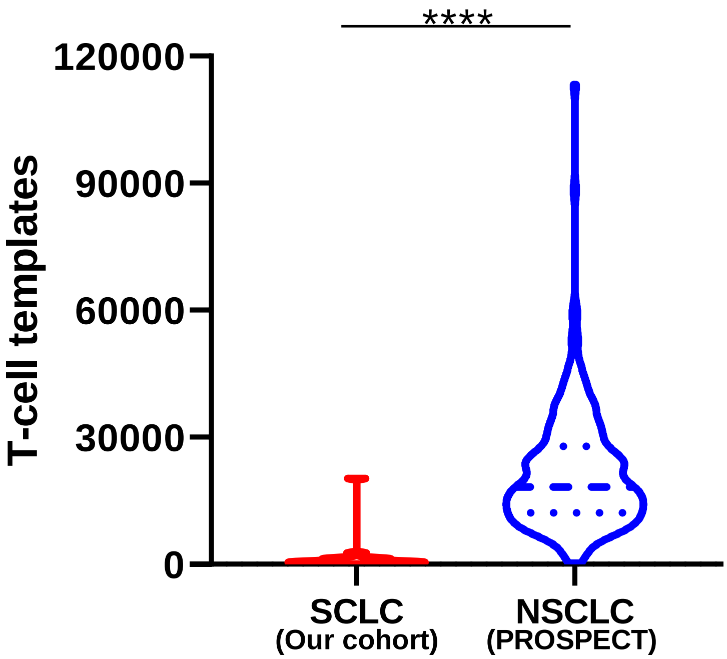

Supplementary Fig. 13 Comparison of T-cell templates between small-cell lung cancer (SCLC) tumors and non-small cell lung cancer (NSCLC) tumors from PROSPECT cohort. T-cell templates were derived from 17 SCLCs (red) *versus* 236 NSCLCs (blue) from the PROSPECT cohort. The difference was evaluated using two-sided Mann-Whitney test. ****p<0.0001. Source data are provided as a Source Data file.

Supplementary Figure 14

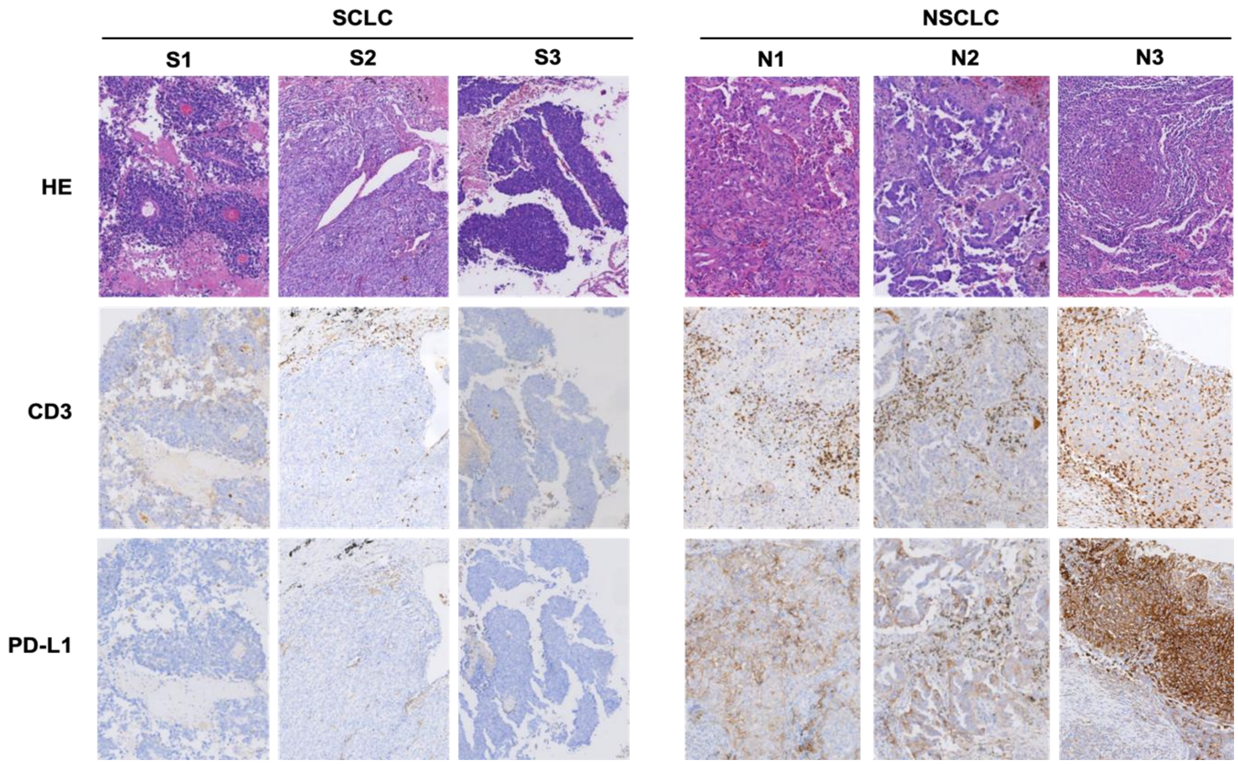

Supplementary Fig. 14 Expression of CD3 in tumor infiltrated lymphocytes (TILs) and programmed death ligand-1 (PD-L1) in small cell lung cancer (SCLC) and non-small cell lung cancer (NSCLC) respectively. S1~3 and N1~3 indicates for three independent cases for SCLC and NSCLC. All the pictures were taken under 100× power phase.

Supplementary Figure 15

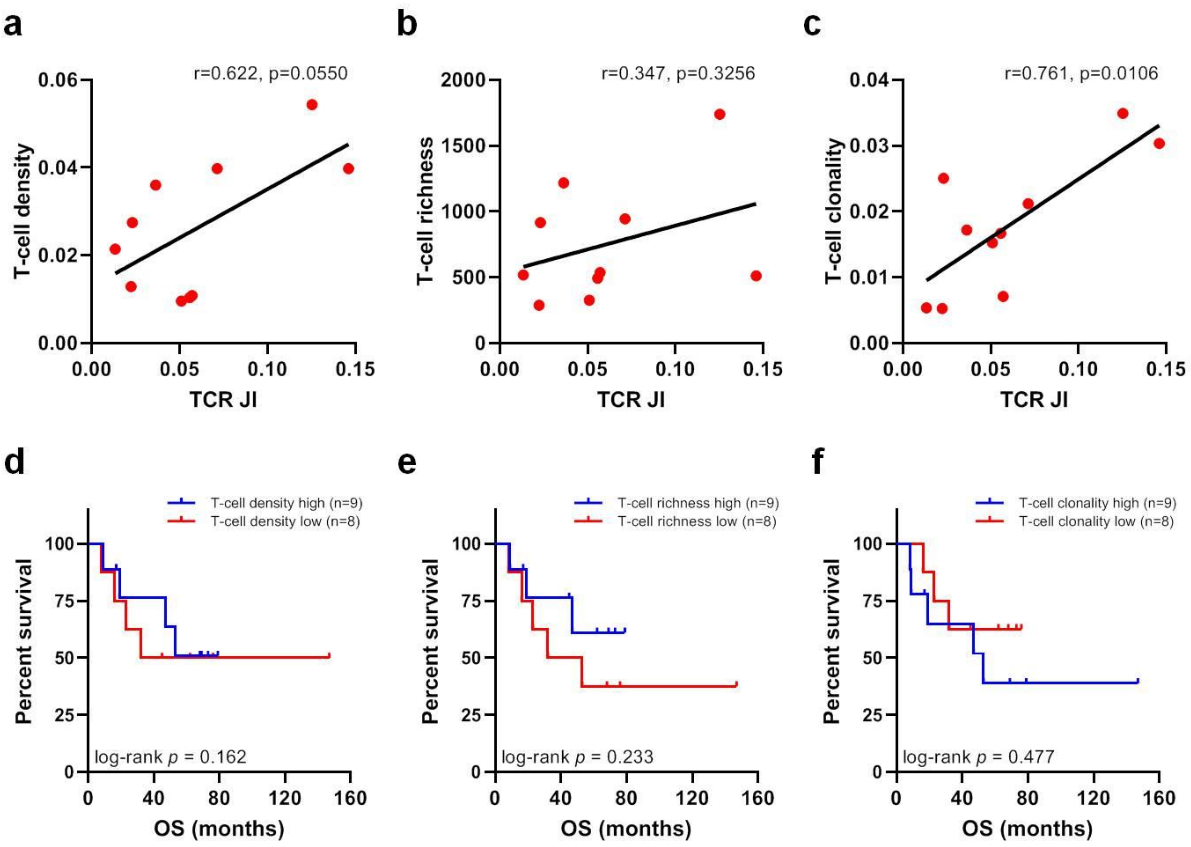

Supplementary Fig. 15 Correlations of T cell receptor (TCR) metrics with TCR Jaccard index (JI) and their associations with overall survival (OS). Positive correlations of TCR JI with T-cell **(a)** density, **(b)** richness and **(c)** clonality in 10 small-cell lung cancer (SCLC) patients with multi-region TCR data available. Associations of OS with T-cell **(d)** density, **(e)** richness and **(f)** clonality in 17 SCLC patients with TCR data available. Two-sided Pearson's correlations were calculated to assess associations. Two-sided log-rank test was used for survival analysis. Source data are provided as a Source Data file.

Supplementary Figure 16

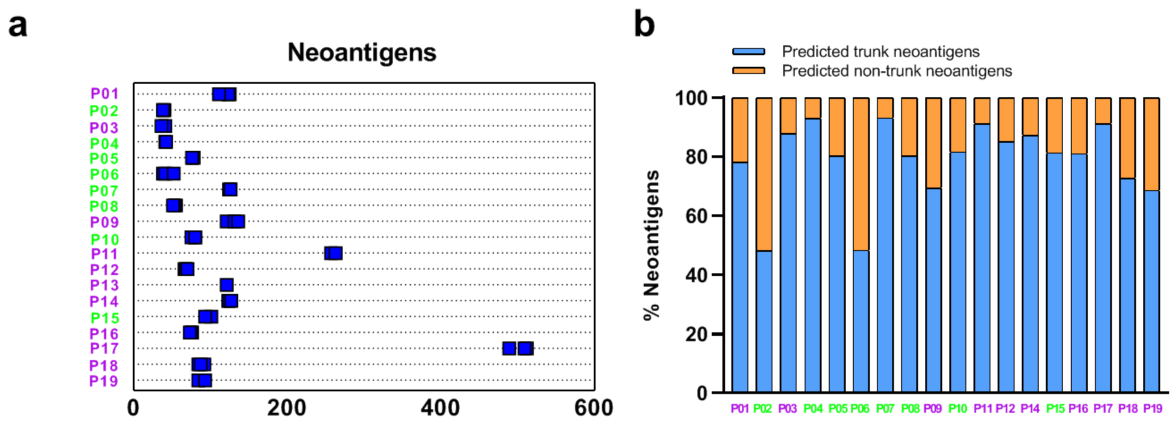

Supplementary Fig. 16 Predicted neoantigen in small-cell lung cancers (SCLCs). (a) Predicted neoantigens in SCLCs. **(b)** Proportion of predicted neoantigens associated with trunk mutations detected in all tumor regions of a given tumor (blue) *versus* non-trunk mutations (orange) mutations in 18 SCLCs with multiregional exome sequencing data available. Purple patient IDs = alive; Green patient IDs = deceased. Source data are provided as a Source Data file.

Supplementary Figure 17

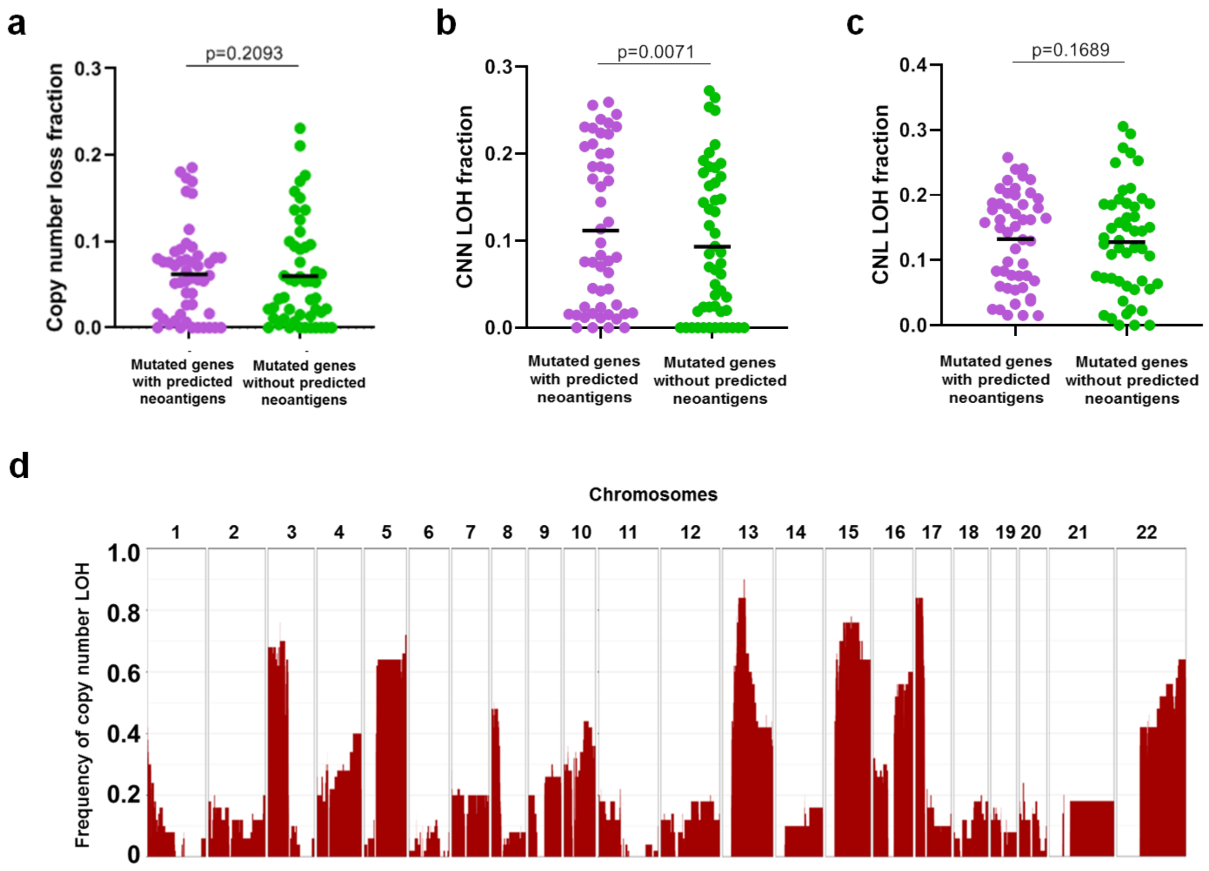

Supplementary Fig. 17 Associations of chromosomal copy number aberrations (CNAs) with loss of neoantigens. (a) copy number loss fraction, **(b)** copy number neutral loss of heterozygosity (CNN-LOH) fraction and **(c)** copy number loss LOH (CNL-LOH) fraction in predicted neoantigen-associated genes (purple) *versus* other mutated genes not associated with predicted neoantigens (green) in 50 small-cell lung cancer (SCLC) tumors. Two-sided Wilcoxon matched pairs signed rank test was used to compare different levels of copy number loss fraction and LOH in each tumor. **(d)** The distribution of LOH at different chromosomal regions in SCLCs. The frequency of LOH was defined as the proportion of samples with LOH among 50 SCLC samples by different chromosomal locations. Source data are provided as a Source Data file.

Supplementary Figure 18

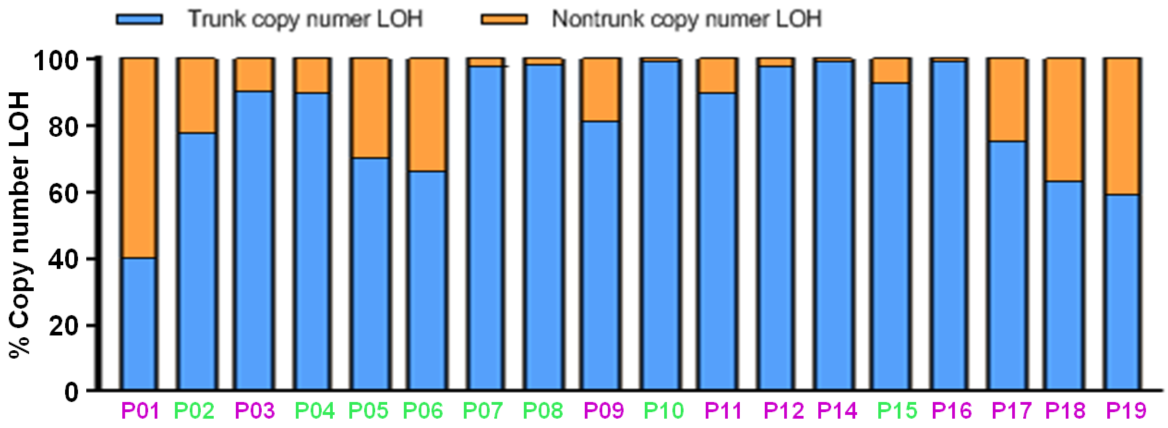

Supplementary Fig. 18 Proportion of trunk *versus* nontrunk LOH of 18 small-cell lung cancers (SCLCs) with multi-region whole exome sequencing (WES) data. Proportion of trunk (blue) and branch (brown) representing loss of heterozygosity (LOH) detected in all tumor regions and some tumor regions from any given tumor. Patient ID: pink = alive; green = deceased. Source data are provided as a Source Data file.

Supplementary Figure 19

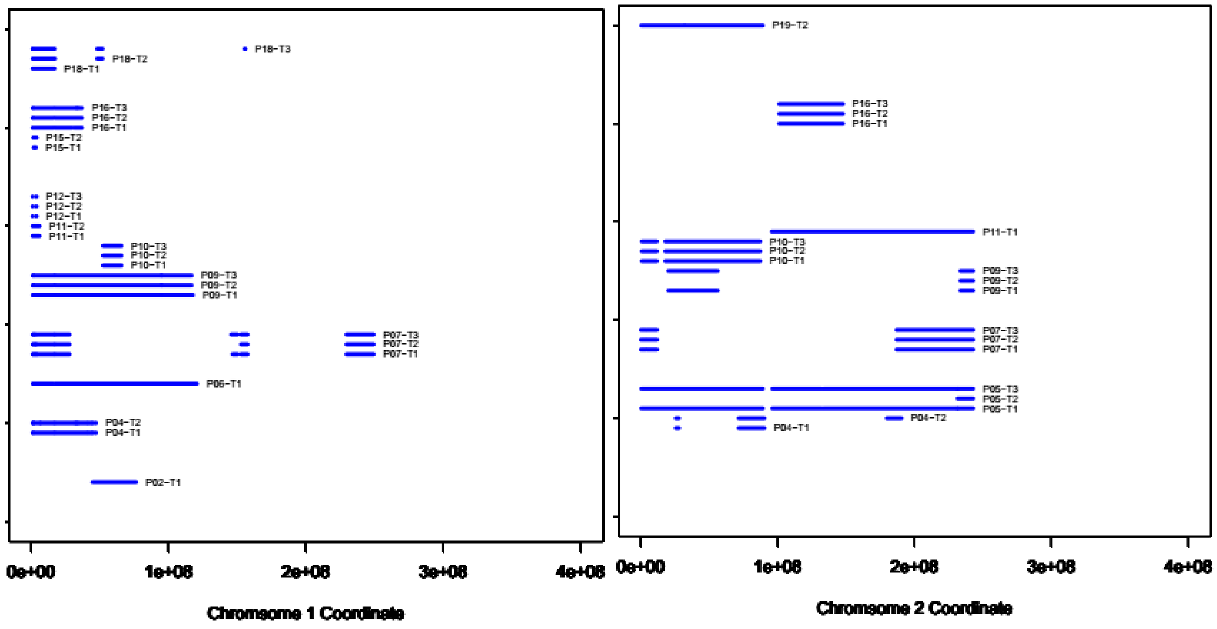

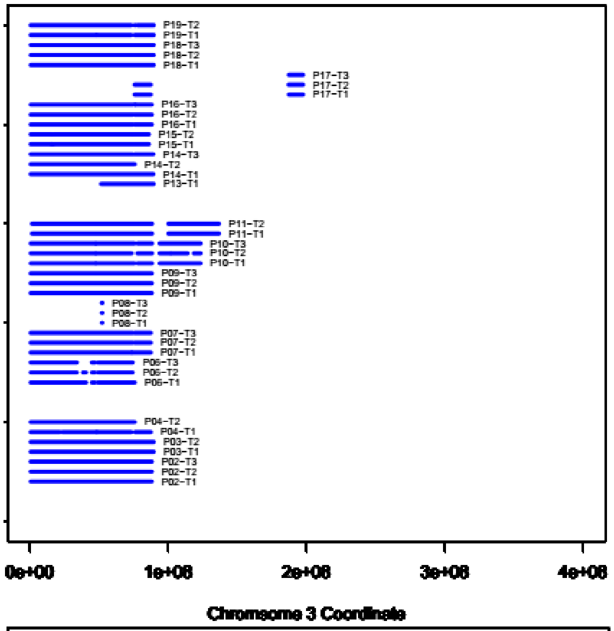
0e+00
1e+08
2e+08
3e+08
4e+08
Chromsome 3 Coordinate

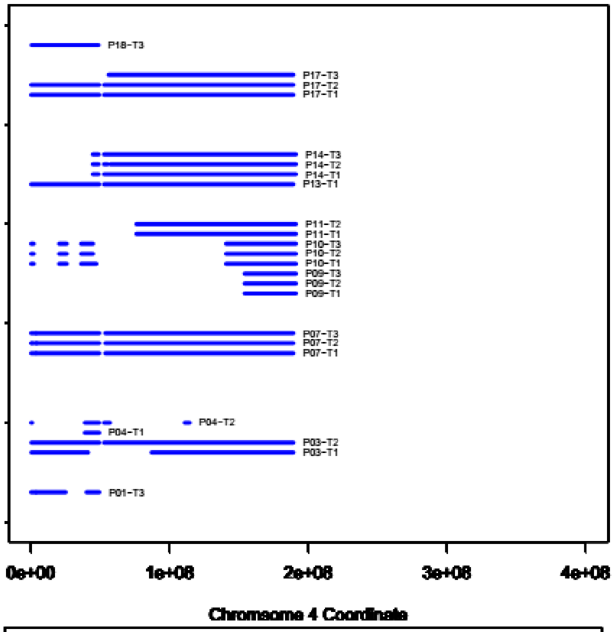
0e+00
1e+08
2e+08
3e+08
4e+08
Chromsome 4 Coordinate

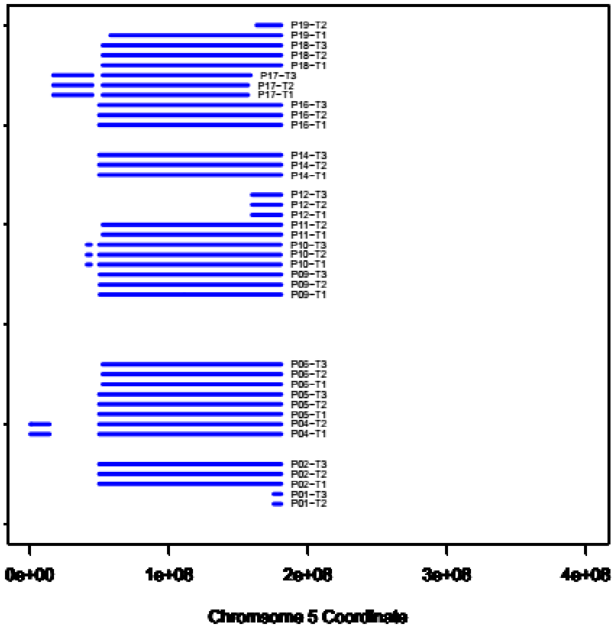
0e+00
1e+08
2e+08
3e+08
4e+08
Chromsome 5 Coordinate

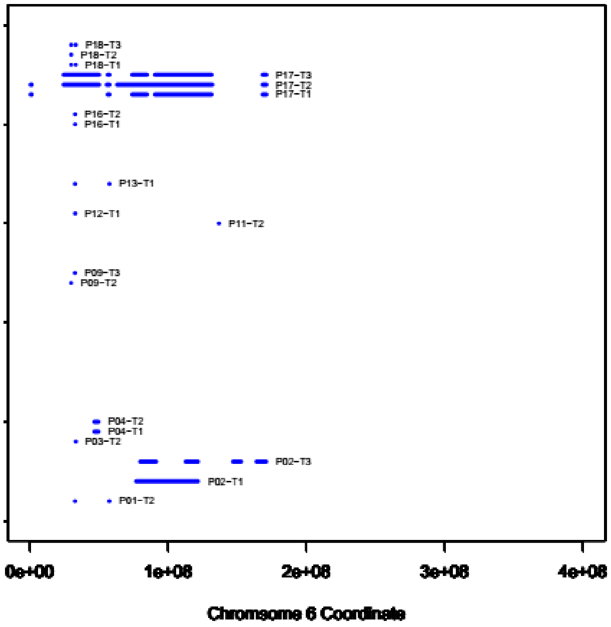
0e+00
1e+08
2e+08
3e+08
4e+08
Chromsome 6 Coordinate

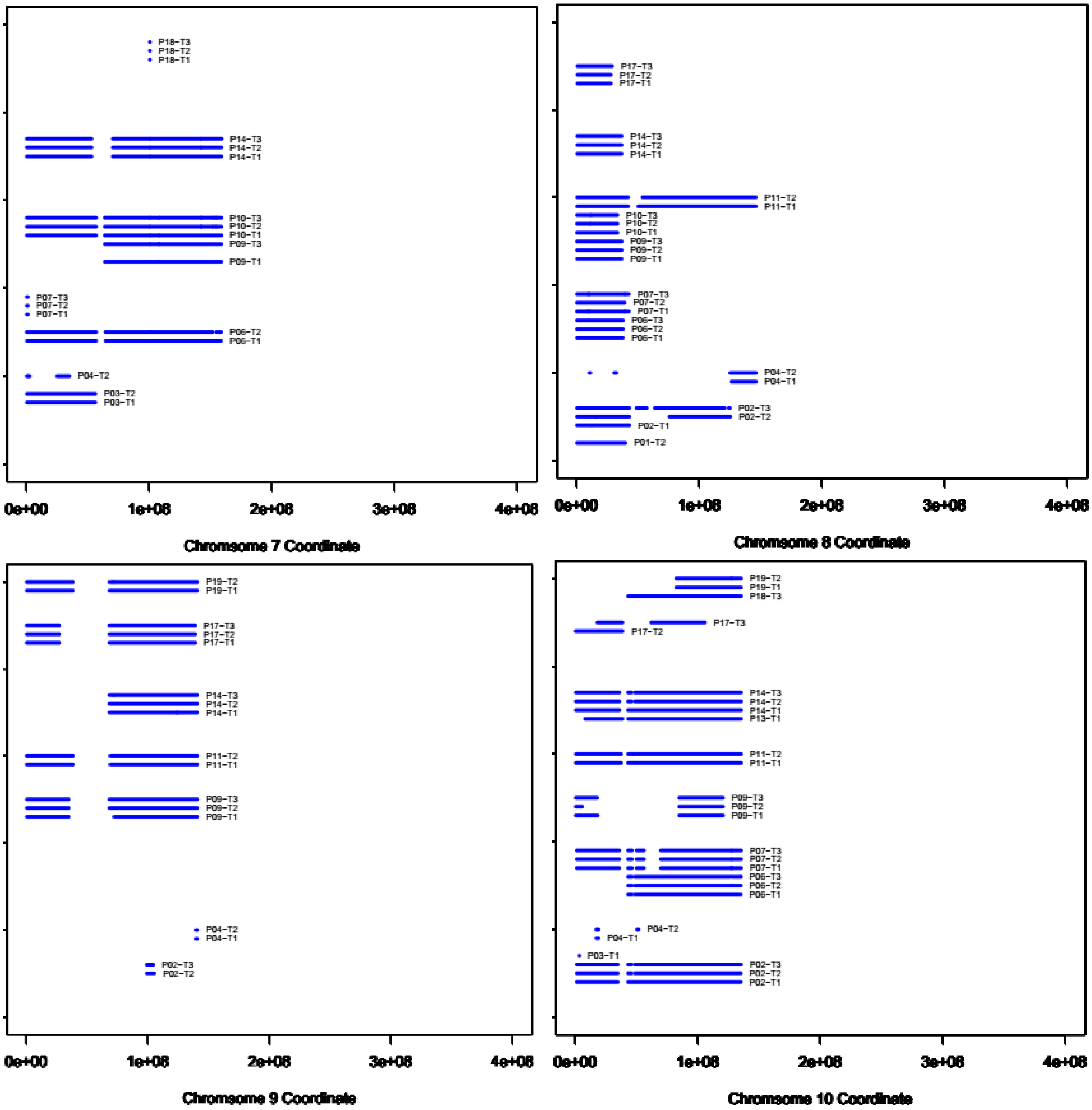
P18−T3
P18−T2
P18−T1
P14−T3
P14−T2
P14−T1
P10−T3
P10−T2
P10−T1
P09−T3
P09−T1
P07−T3
P07−T2
P07−T1
P06−T2
P06−T1
P04−T2
P03−T2
P03−T1
0e+00
1e+08
2e+08
3e+08
4e+08
Chromosome 7 Coordinate
P17−T3
P17−T2
P17−T1
P14−T3
P14−T2
P14−T1
P11−T2
P11−T1
P10−T3
P10−T2
P10−T1
P09−T3
P09−T2
P09−T1
P07−T3
P07−T2
P07−T1
P06−T3
P06−T2
P06−T1
P04−T2
P04−T1
P02−T3
P02−T2
P02−T1
P01−T2
0e+00
1e+08
2e+08
3e+08
4e+08
Chromosome 8 Coordinate
P19−T2
P19−T1
P17−T3
P17−T2
P17−T1
P14−T3
P14−T2
P14−T1
P11−T2
P11−T1
P09−T3
P09−T2
P09−T1
P04−T2
P04−T1
P02−T3
P02−T2
0e+00
1e+08
2e+08
3e+08
4e+08
Chromosome 9 Coordinate
P19−T2
P19−T1
P18−T3
P17−T3
P17−T2
P14−T3
P14−T2
P14−T1
P13−T1
P11−T2
P11−T1
P09−T3
P09−T2
P09−T1
P07−T3
P07−T2
P07−T1
P06−T3
P06−T2
P06−T1
P04−T2
P04−T1
P03−T1
P02−T3
P02−T2
P02−T1
0e+00
1e+08
2e+08
3e+08
4e+08
Chromosome 10 Coordinate

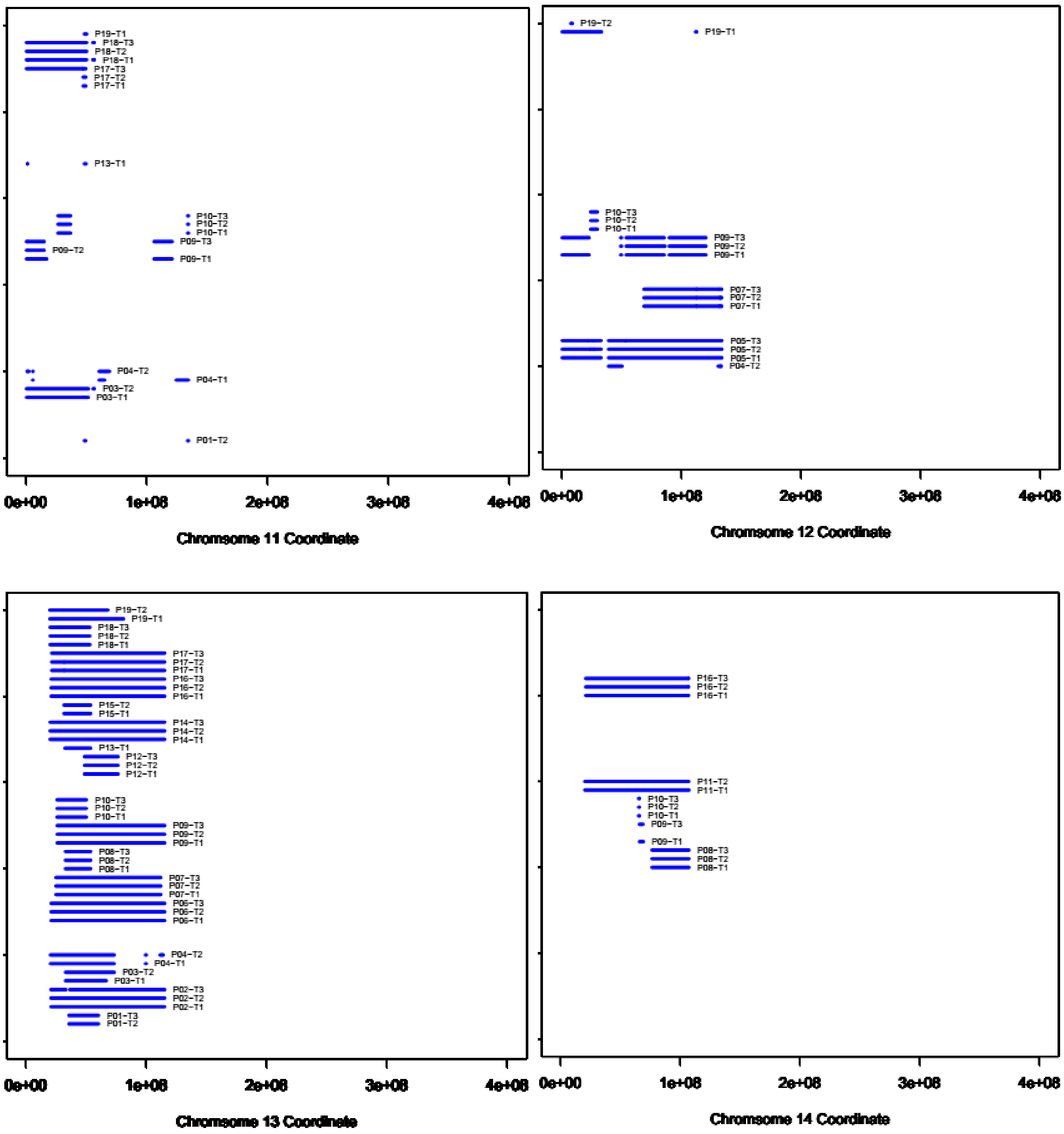
P19−T1
P18−T3
P18−T2
P18−T1
P17−T3
P17−T2
P17−T1
P13−T1
P10−T3
P10−T2
P10−T1
P09−T3
P09−T2
P09−T1
P04−T2
P04−T1
P03−T2
P03−T1
P01−T2
0e+00
1e+08
2e+08
3e+08
4e+08
Chromsome 11 Coordinate
P19−T2
P19−T1
P10−T3
P10−T2
P10−T1
P09−T3
P09−T2
P09−T1
P07−T3
P07−T2
P07−T1
P05−T3
P05−T2
P05−T1
P04−T2
0e+00
1e+08
2e+08
3e+08
4e+08
Chromsome 12 Coordinate
P19−T2
P19−T1
P18−T3
P18−T2
P18−T1
P17−T3
P17−T2
P17−T1
P16−T3
P16−T2
P16−T1
P15−T2
P15−T1
P14−T3
P14−T2
P14−T1
P13−T1
P12−T3
P12−T2
P12−T1
P10−T3
P10−T2
P10−T1
P09−T3
P09−T2
P09−T1
P08−T3
P08−T2
P08−T1
P07−T3
P07−T2
P07−T1
P06−T3
P06−T2
P06−T1
P04−T2
P04−T1
P03−T2
P03−T1
P02−T3
P02−T2
P02−T1
P01−T3
P01−T2
0e+00
1e+08
2e+08
3e+08
4e+08
Chromsome 13 Coordinate
P16−T3
P16−T2
P16−T1
P11−T2
P11−T1
P10−T3
P10−T2
P10−T1
P09−T3
P09−T1
P08−T3
P08−T2
P08−T1
0e+00
1e+08
2e+08
3e+08
4e+08
Chromsome 14 Coordinate

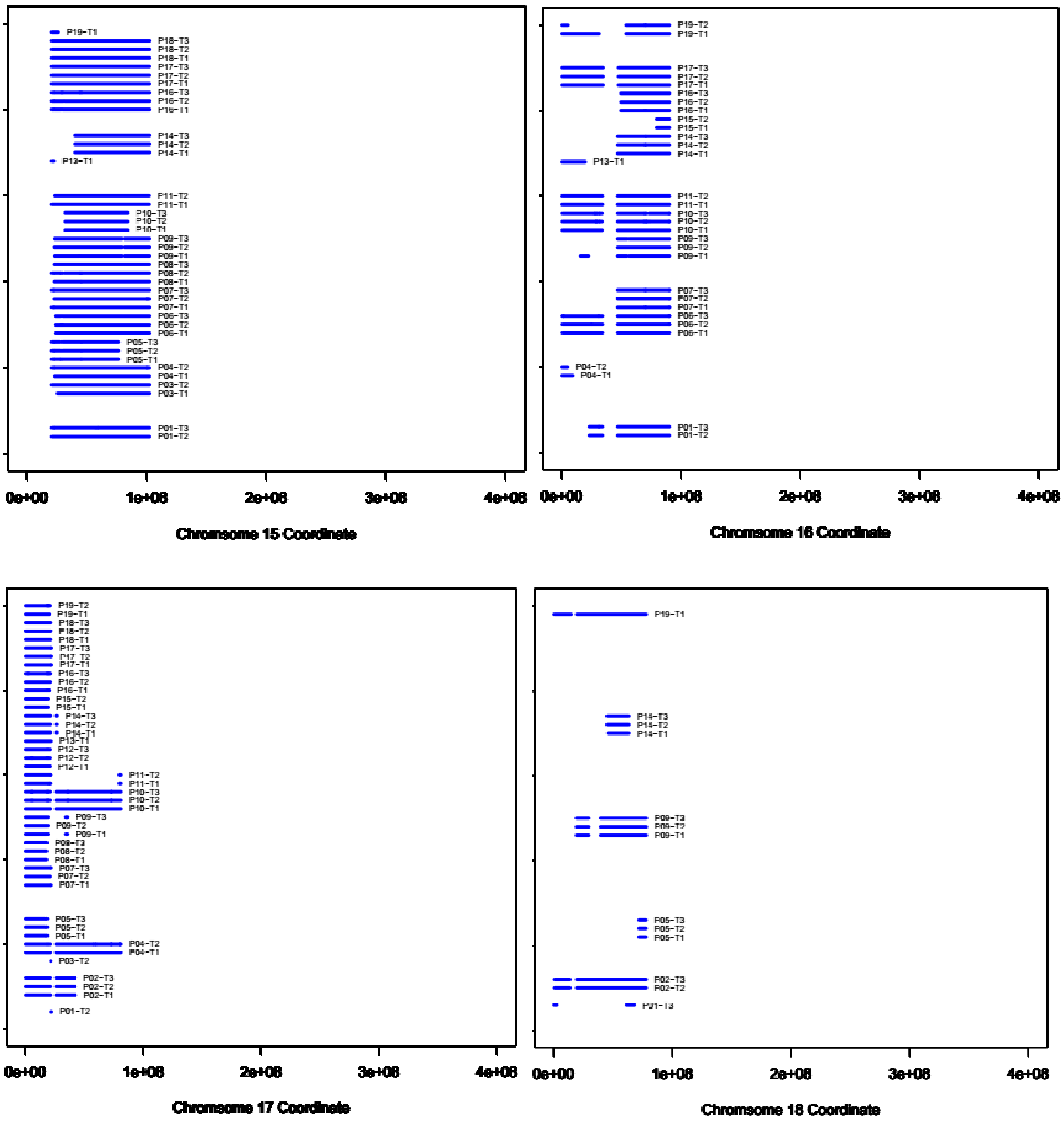
0e+00
1e+08
2e+08
3e+08
4e+08
Chromsome 15 Coordinate
Chromsome 16 Coordinate
Chromsome 17 Coordinate
Chromsome 18 Coordinate

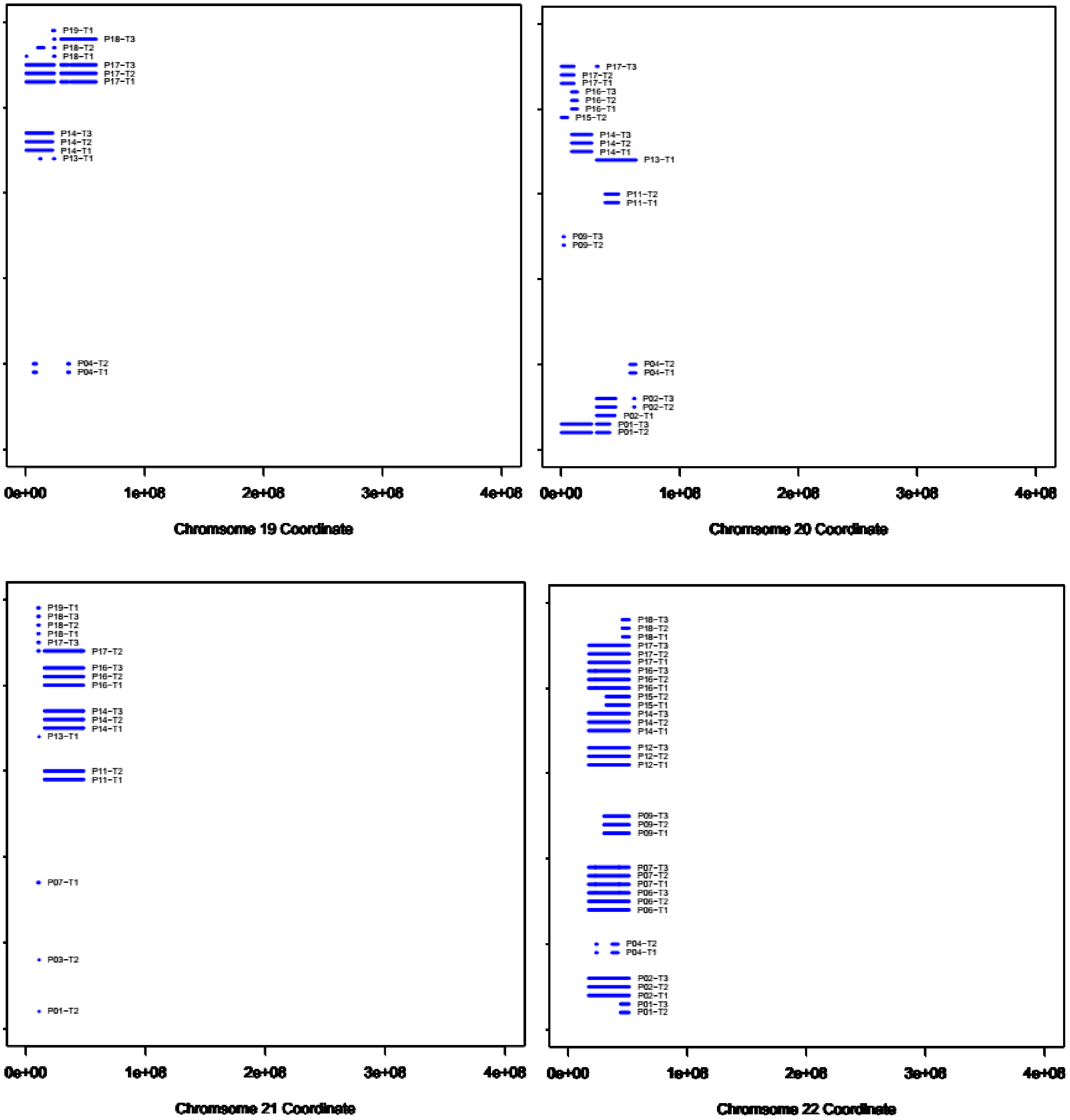

Supplementary Fig. 19 Distribution of LOH events in different specimens across the genome (chromosome 1-22). The loss of heterozygosity (LOH) events of various size were shown as blue bars and sample IDs were shown on the right.

Supplementary Figure 20

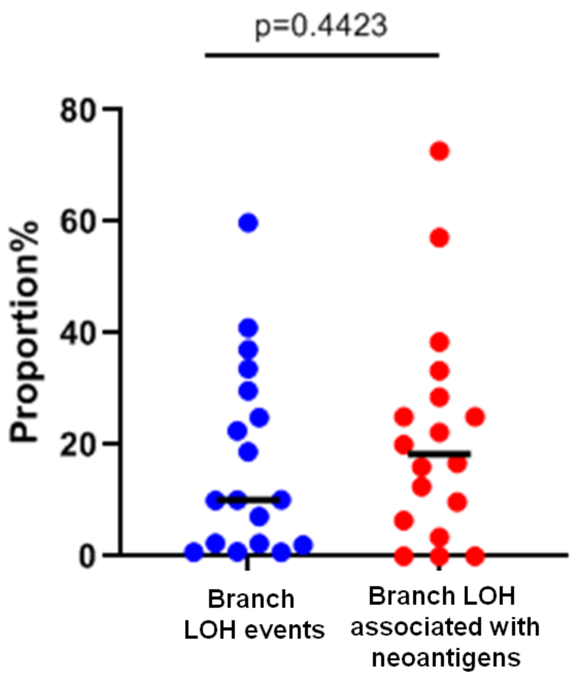

Supplementary Fig. 20 Proportion of LOH events that are present as branch (present in some but not all tumor regions within the tumors) versus branch LOH associated with predicted neoantigens in small-cell lung cancers (SCLCs). Each dot represents one tumor. Blue: the proportion of branch loss of heterozygosity (LOH) among all LOH events in 18 patients. Red: the proportion of branch LOH associated with neoantigens in 18 patients. Two-sided Wilcoxon matched pairs signed rank test was used. Source data are provided as a Source Data file.

Supplementary Figure 21

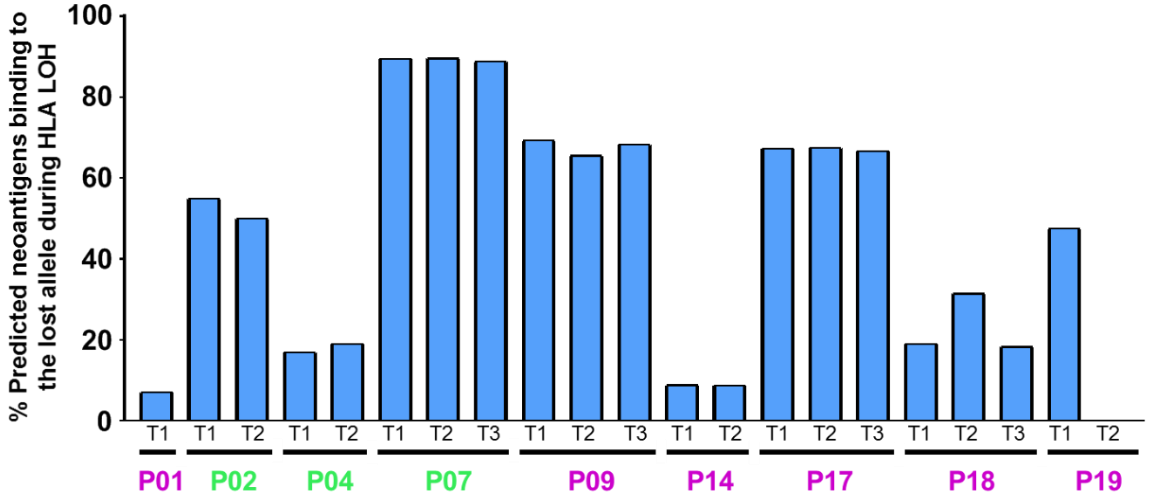

Supplementary Fig. 21 Proportion of predicted neoantigens binding to the lost allele during HLA LOH in small-cell lung cancers (SCLCs). Proportion of predicted neoantigens binding to the lost allele was calculated in 21 SCLC samples with HLA loss of heterozygosity (LOH). Purple patient IDs = alive; Green patient IDs = deceased. Source data are provided as a Source Data file.

Supplementary Figure 22

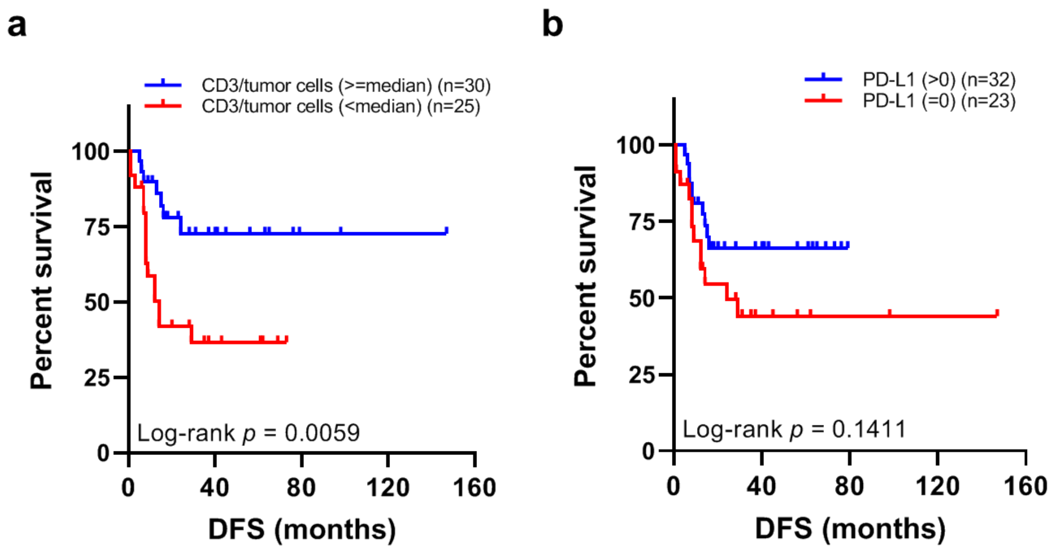

Supplementary Fig. 22 Association of tumor purity, CD3 tumor infiltrating lymphocytes (TILs) and PD-L1 expressions with disease-free survival (DFS). (a) DFS in patients with tumors of higher (⩾median, blue) CD3+ TILs versus patients with tumors of lower (<median, red) CD3+ TILs. **(b)** DFS in patients with tumors of positive (above 0, blue) programmed death ligand-1 (PD-L1) versus patients with tumors of negative (equal 0, red) PD-L1 expression. Two-sided log-rank test was used for survival analysis. Source data are provided as a Source Data file.

Supplementary Figure 23

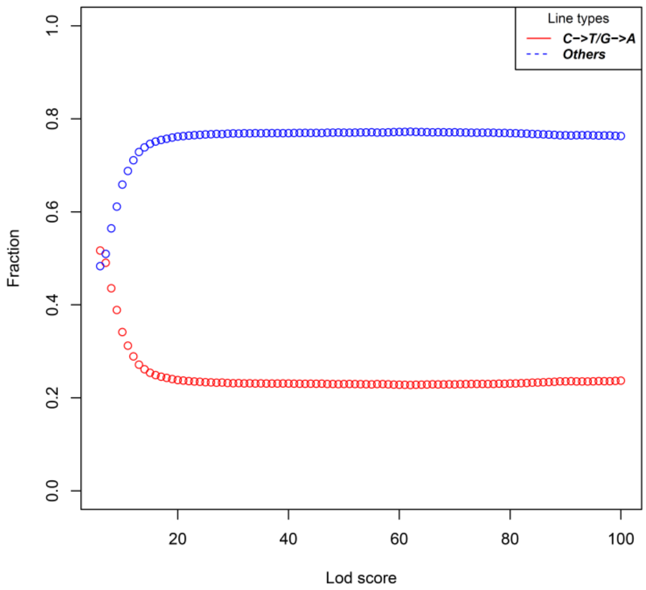

Supplementary Fig. 23 The distribution of the proportion of C>T/G>A transitions by LOD scores in 50 small-cell lung cancer (SCLC) specimens. X-axis shows the log odds (LOD) scores of mutation calls and Y-axis represents the proportion of C>T/G>A transitions among all mutations of any given LOD threshold.

Supplementary Figure 24

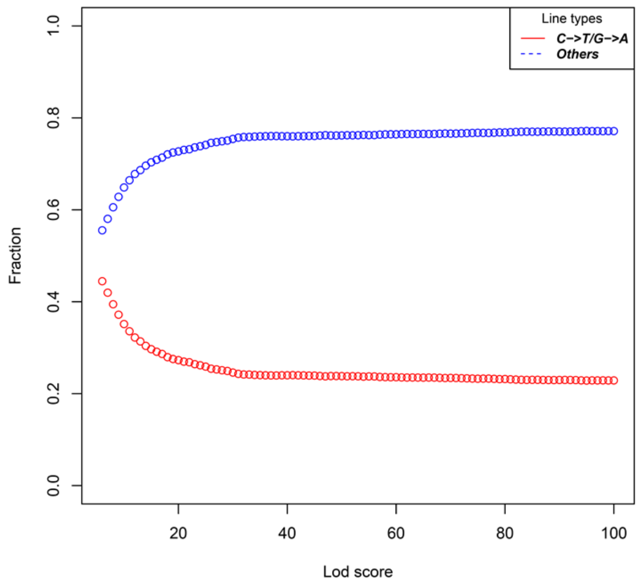

Supplementary Fig. 24 The distribution of the proportion of C>T/G>A transitions by LOD scores in small-cell lung cancer (SCLC) at tumor level. The mutations were called on merged bam files from different tumor regions within the same tumors from 19 SCLC patients. X-axis shows the log odds (LOD) scores of mutation calls and Y-axis represents the proportion of C>T/G>A transitions among all mutations of any given LOD threshold.
