# Supplementary material for: Cold and heterogeneous T cell repertoire is associated with copy number aberrations and loss of immune genes in small-cell lung cancer
Source: Nat Commun. 2021 Nov 17;12:6655. doi: 10.1038/s41467-021-26821-8 (PMC8599854; doi:10.1038/s41467-021-26821-8)
Supplement: Supplementary file 2 — Description of Additional Supplementary Files [file 41467_2021_26821_MOESM2_ESM.pdf]

Title: Supplementary Data 1.

Description: Clinical characteristics and key immunogenomic features of small-cell lung cancers (SCLCs) (n=67) and non-small cell lung cancers (NSCLCs) (n=68)

Title: Supplementary Data 2.

Description: Somatic mutations in 50 small-cell lung cancer (SCLC) tumor specimens

Title: Supplementary Data 3.

Description: Pathological assessment of multi-region small-cell lung cancer (SCLC) samples (n=50)
