# Supplementary material for: Cold and heterogeneous T cell repertoire is associated with copy number aberrations and loss of immune genes in small-cell lung cancer
Source: Nat Commun. 2021 Nov 17;12:6655. doi: 10.1038/s41467-021-26821-8 (PMC8599854; doi:10.1038/s41467-021-26821-8)
Supplement: Supplementary file 6 — Reporting Summary [file 41467_2021_26821_MOESM6_ESM.pdf]

nature research

Corresponding author(s): Jianjun Zhang

Last updated by author(s): Oct 21, 2021

Reporting Summary

Nature Research wishes to improve the reproducibility of the work that we publish. This form provides structure for consistency and transparency in reporting. For further information on Nature Research policies, see our Editorial Policies and the Editorial Policy Checklist.

Statistics

For all statistical analyses, confirm that the following items are present in the figure legend, table legend, main text, or Methods section.

n/a	Confirmed	
☐	☒	The exact sample size (n) for each experimental group/condition, given as a discrete number and unit of measurement
☐	☒	A statement on whether measurements were taken from distinct samples or whether the same sample was measured repeatedly
☐	☒	The statistical test(s) used AND whether they are one- or two-sided<br>*Only common tests should be described solely by name; describe more complex techniques in the Methods section.*
☐	☒	A description of all covariates tested
☐	☒	A description of any assumptions or corrections, such as tests of normality and adjustment for multiple comparisons
☐	☒	A full description of the statistical parameters including central tendency (e.g. means) or other basic estimates (e.g. regression coefficient) AND variation (e.g. standard deviation) or associated estimates of uncertainty (e.g. confidence intervals)
☐	☒	For null hypothesis testing, the test statistic (e.g. F, t, r) with confidence intervals, effect sizes, degrees of freedom and P value noted<br>*Give P values as exact values whenever suitable.*
☒	☐	For Bayesian analysis, information on the choice of priors and Markov chain Monte Carlo settings
☒	☐	For hierarchical and complex designs, identification of the appropriate level for tests and full reporting of outcomes
☐	☒	Estimates of effect sizes (e.g. Cohen's d, Pearson's r), indicating how they were calculated

Our web collection on statistics for biologists contains articles on many of the points above.

Software and code

Policy information about availability of computer code

Data collection	No software was used for data collection. A total of 67 patients with SCLC and 68 patients with NSCLC were included.

Data analysis

The BWA aligner (bwa-0.7.5a) was applied to map the raw reads to the human hg19 reference genome (UCSC genome browser: genome.ucsc.edu). The Picard (v1.112, http://broadinstitute.github.io/picard/) ''MarkDuplicates'' module was applied to mark the duplicate reads. Then the ''IndelRealigner'' and ''BaseRecalibrator'' modules of the Genome Analysis Toolkit were applied to perform indel realignment and base quality recalibration. Mutect (v1.1.4) was applied to identify somatic single nucleotide variants (SNVs) and small insertions/ deletions. Tumor contents and major/minor copy number changes were then estimated by Sequenza (v2.1.2). Pyclone-VI was applied to estimate the clonal status of each mutation. Copy numbers of somatic mutations were inferred by integrating integer copy numbers determined by Sequenza (v2.1.2) on single sample basis. Multistate discrete-characters Wagner parsimony method in PHYLIP (Phylogeny Inference Package) (version 3.695) was used to generate phylogenic tree. The R package "DeconstructSigs" package (version 1.8.0) was applied to estimate the proportions of 30 COSMIC mutational signatures (http://cancer.sanger.ac.uk/cosmic/signatures). ExomeCNV (version 1.4) was applied to infer somatic copy-number alteration. CNTools package (version 1.49.0) was used to segment DNA copy number profiles at the gene level. The binding affinity with patient-restricted MHC Class I molecules of all possible 9- and 10-mer peptides was evaluated with the NetMHC3.4 algorithm based on patient HLA-A, HLA-B, and HLA-C alleles. HLA allele was predicted by POLYSOLVER (version 1.2.0). Candidate peptides were considered HLA binders when IC50<500 nM. Class I HLA alleles for each HLA gene was inferred from germline DNA by POLYSOLVER (version 1.2.0) using a two-step Bayesian classification approach, which takes into account the base qualities of aligned reads, observed insert sizes, as well as the ethnicity-dependent prior probabilities of each allele. Tumor purity and ploidy were then estimated using Sequenza (v2.1.2), and LOHHLA (Loss Of Heterozygosity in Human Leukocyte Antigen) algorithm was applied to detect allele-specific HLA loss in each tumor sample. The segment copy number file (predicted by Sequenza (v2.1.2)) was used to evaluate whether a gene showing evidence of copy-number-neutral LOH (CNN-LOH) or copy-number-loss LOH (CNL-LOH). The estimated T-cell fraction was calculated by MCPcounter (version 1.0.0). The immune score was calculated with the R package "ESTIMATE" (https://sourceforge.net/projects/ estimateproject/). TCR sequencing analysis was performed in R version 3.2 in ImmunoSEQ ANALYZER (ANALYSES 3.0). Graphs were generated with GraphPad Prism 8.0 (La Jolla, CA).

For manuscripts utilizing custom algorithms or software that are central to the research but not yet described in published literature, software must be made available to editors and reviewers. We strongly encourage code deposition in a community repository (e.g. GitHub). See the Nature Research guidelines for submitting code & software for further information.

Data

Policy information about availability of data

All manuscripts must include a data availability statement. This statement should provide the following information, where applicable:

- Accession codes, unique identifiers, or web links for publicly available datasets
- A list of figures that have associated raw data
- A description of any restrictions on data availability

The whole exome sequencing (WES) data in this study have been deposited in the European Bioinformatics Institute European Genome–phenome Archive (EGA) (accession number: EGAS00001005087) through controlled access. T cell receptor (TCR) sequencing data in this study are available through the immuneACCESS platform (10.21417/MC2021NC; https://clients.adaptivebiotech.com/pub/chen-2021-nc). To protect patient privacy, researchers interested in the WES data need to apply via data access committee (DAC) by contacting Dr. Jianjun Zhang at Jzhang20@mdanderson.org, which will grant all reasonable requests. TCR sequencing data has been shared as public data. The WES data of PROSPECT cohort used in this study are available in the EGA database under accession soce EGAS00001004026. TCR sequencing data of PROSPECT cohort are available through the immuneACCESS platform (10.21417/AR2019NC https:// clients.adaptivebiotech.com/pub/reuben-2019-natcomms). Sequencing data of George cohort used in this study are available in the EGA database under the accession code EGAS00001000925. Sequencing data of TRACERx cohort used in this study are available in the EGA database under the accession code EGAS00001002247. Sequencing data of TCGA used in this study is available in cgHub and the Data Coordinating Center (DCC) for public access (http:// cancergenome.nih.gov/, https://cghub.ucsc.edu/ and https://www.cancer.gov/about-nci/organization/ccg/research/structural-genomics/tcga). All other data may be found within the Article file, Supplementary information or Source Data file.

Field-specific reporting

Please select the one below that is the best fit for your research. If you are not sure, read the appropriate sections before making your selection.

☒ Life sciences ☐ Behavioural & social sciences ☐ Ecological, evolutionary & environmental sciences

For a reference copy of the document with all sections, see nature.com/documents/nr-reporting-summary-flat.pdf

Life sciences study design

All studies must disclose on these points even when the disclosure is negative.

Sample size	Sample size was not determined before the study, rather based on sample availability. The specimens were collected through international collaborations. All available samples were subjected to the analysis.
Data exclusions	No data were excluded from the analyses, as all samples was already subjected to rigorous QC by the sequence core. Since only a subset of samples are available for each analysis, we did analysis based on sample availability.
Replication	Due to scarcity of resected specimens from small-cell lung cancer, all available samples were subjected to the analysis. Replication was not feasible.
Randomization	This is not a study involving clinical trial, randomization does not apply to this study. This is not a study involving clinical trial, randomization does not apply to this study. Due to scarcity of resected specimens from small-cell lung cancers, all available samples were subjected to immunogenomic profiling and the corresponding analysis.

Blinding	This is not a study involving clinical trial, and due to scarcity of resected specimens from small-cell lung cancers, blinding does not apply to this study. And the analyst doesn't know the clinical information of the patients.

Behavioural & social sciences study design

All studies must disclose on these points even when the disclosure is negative.

Study description	*Briefly describe the study type including whether data are quantitative, qualitative, or mixed-methods (e.g. qualitative cross-sectional, quantitative experimental, mixed-methods case study).*
Research sample	*State the research sample (e.g. Harvard university undergraduates, villagers in rural India) and provide relevant demographic information (e.g. age, sex) and indicate whether the sample is representative. Provide a rationale for the study sample chosen. For studies involving existing datasets, please describe the dataset and source.*
Sampling strategy	*Describe the sampling procedure (e.g. random, snowball, stratified, convenience). Describe the statistical methods that were used to predetermine sample size OR if no sample-size calculation was performed, describe how sample sizes were chosen and provide a rationale for why these sample sizes are sufficient. For qualitative data, please indicate whether data saturation was considered, and what criteria were used to decide that no further sampling was needed.*
Data collection	*Provide details about the data collection procedure, including the instruments or devices used to record the data (e.g. pen and paper, computer, eye tracker, video or audio equipment) whether anyone was present besides the participant(s) and the researcher, and whether the researcher was blind to experimental condition and/or the study hypothesis during data collection.*
Timing	*Indicate the start and stop dates of data collection. If there is a gap between collection periods, state the dates for each sample cohort.*
Data exclusions	*If no data were excluded from the analyses, state so OR if data were excluded, provide the exact number of exclusions and the rationale behind them, indicating whether exclusion criteria were pre-established.*
Non-participation	*State how many participants dropped out/declined participation and the reason(s) given OR provide response rate OR state that no participants dropped out/declined participation.*
Randomization	*If participants were not allocated into experimental groups, state so OR describe how participants were allocated to groups, and if allocation was not random, describe how covariates were controlled.*

Ecological, evolutionary & environmental sciences study design

All studies must disclose on these points even when the disclosure is negative.

Study description	*Briefly describe the study. For quantitative data include treatment factors and interactions, design structure (e.g. factorial, nested, hierarchical), nature and number of experimental units and replicates.*
Research sample	*Describe the research sample (e.g. a group of tagged Passer domesticus, all Stenocereus thurberi within Organ Pipe Cactus National Monument), and provide a rationale for the sample choice. When relevant, describe the organism taxa, source, sex, age range and any manipulations. State what population the sample is meant to represent when applicable. For studies involving existing datasets, describe the data and its source.*
Sampling strategy	*Note the sampling procedure. Describe the statistical methods that were used to predetermine sample size OR if no sample-size calculation was performed, describe how sample sizes were chosen and provide a rationale for why these sample sizes are sufficient.*
Data collection	*Describe the data collection procedure, including who recorded the data and how.*
Timing and spatial scale	*Indicate the start and stop dates of data collection, noting the frequency and periodicity of sampling and providing a rationale for these choices. If there is a gap between collection periods, state the dates for each sample cohort. Specify the spatial scale from which the data are taken*
Data exclusions	*If no data were excluded from the analyses, state so OR if data were excluded, describe the exclusions and the rationale behind them, indicating whether exclusion criteria were pre-established.*
Reproducibility	*Describe the measures taken to verify the reproducibility of experimental findings. For each experiment, note whether any attempts to repeat the experiment failed OR state that all attempts to repeat the experiment were successful.*
Randomization	*Describe how samples/organisms/participants were allocated into groups. If allocation was not random, describe how covariates were controlled. If this is not relevant to your study, explain why.*
Blinding	*Describe the extent of blinding used during data acquisition and analysis. If blinding was not possible, describe why OR explain why blinding was not relevant to your study.*

Did the study involve field work? ☐ Yes ☐ No

Field work, collection and transport

Field conditions	*Describe the study conditions for field work, providing relevant parameters (e.g. temperature, rainfall).*
Location	*State the location of the sampling or experiment, providing relevant parameters (e.g. latitude and longitude, elevation, water depth).*
Access & import/export	*Describe the efforts you have made to access habitats and to collect and import/export your samples in a responsible manner and in compliance with local, national and international laws, noting any permits that were obtained (give the name of the issuing authority, the date of issue, and any identifying information).*
Disturbance	*Describe any disturbance caused by the study and how it was minimized.*

Reporting for specific materials, systems and methods

We require information from authors about some types of materials, experimental systems and methods used in many studies. Here, indicate whether each material, system or method listed is relevant to your study. If you are not sure if a list item applies to your research, read the appropriate section before selecting a response.

Materials & experimental systems

n/a	Involved in the study	
☐	☒	Antibodies
☒	☐	Eukaryotic cell lines
☒	☐	Palaeontology and archaeology
☒	☐	Animals and other organisms
☐	☒	Human research participants
☐	☒	Clinical data
☒	☐	Dual use research of concern

Methods

n/a	Involved in the study	
☒	☐	ChIP-seq
☒	☐	Flow cytometry
☒	☐	MRI-based neuroimaging

Antibodies

Antibodies used	PD-L1 IHC testing was performed using the PD-L1 clone 22C3 pharmDx kit and Dako Automated Link 48 platform (Cat No. SK006, Agilent Technologies/Dako, Carpinteria, CA, USA; This antibody was provided by Merck & Co., Inc.). Slides stained with CD3 were labeled by a mouse anti-CD3 monoclonal antibody (BOND™ Ready-to-Use Primary Antibody CD3 (LN10) (no dilution), Cat No. PA0553, Leica Biosystems, Newcastle Upon Tyne, UK; https://shop.leicabiosystems.com/zh-cn/ihc-ish/ihc-primary-antibodies/pid-cd3) at a working solution and incubated on an autostainer (Leica BOND-III, Leica Biosystems, Newcastle Upon Tyne, UK).
Validation	PD-L1: PD-L1 clone 22C3 pharmDx kit and Dako Automated Link 48 platform (Cat No. SK006, Agilent Technologies/Dako, Carpinteria, CA, USA; This antibody was provided by Merck & Co., Inc.).<br>CD3: labeled by a mouse anti-CD3 monoclonal antibody (BOND™ Ready-to-Use Primary Antibody CD3 (LN10), Cat No. PA0553, Leica Biosystems, Newcastle Upon Tyne, UK; https://shop.leicabiosystems.com/zh-cn/ihc-ish/ihc-primary-antibodies/pid-cd3) at a working solution and incubated on an autostainer (Leica BOND-III, Leica Biosystems, Newcastle Upon Tyne, UK).

Eukaryotic cell lines

Policy information about cell lines

Cell line source(s)	*State the source of each cell line used.*
Authentication	*Describe the authentication procedures for each cell line used OR declare that none of the cell lines used were authenticated.*
Mycoplasma contamination	*Confirm that all cell lines tested negative for mycoplasma contamination OR describe the results of the testing for mycoplasma contamination OR declare that the cell lines were not tested for mycoplasma contamination.*
Commonly misidentified lines<br>(See ICLAC register)	*Name any commonly misidentified cell lines used in the study and provide a rationale for their use.*

Palaeontology and Archaeology

Specimen provenance	*Provide provenance information for specimens and describe permits that were obtained for the work (including the name of the issuing authority, the date of issue, and any identifying information).*
Specimen deposition	*Indicate where the specimens have been deposited to permit free access by other researchers.*

Dating methods | *If new dates are provided, describe how they were obtained (e.g. collection, storage, sample pretreatment and measurement), where they were obtained (i.e. lab name), the calibration program and the protocol for quality assurance OR state that no new dates are provided.*

☐ Tick this box to confirm that the raw and calibrated dates are available in the paper or in Supplementary Information.

Ethics oversight | *Identify the organization(s) that approved or provided guidance on the study protocol, OR state that no ethical approval or guidance was required and explain why not.*

Note that full information on the approval of the study protocol must also be provided in the manuscript.

Animals and other organisms

Policy information about studies involving animals; ARRIVE guidelines recommended for reporting animal research

Laboratory animals | *For laboratory animals, report species, strain, sex and age OR state that the study did not involve laboratory animals.*

Wild animals | *Provide details on animals observed in or captured in the field; report species, sex and age where possible. Describe how animals were caught and transported and what happened to captive animals after the study (if killed, explain why and describe method; if released, say where and when) OR state that the study did not involve wild animals.*

Field-collected samples | *For laboratory work with field-collected samples, describe all relevant parameters such as housing, maintenance, temperature, photoperiod and end-of-experiment protocol OR state that the study did not involve samples collected from the field.*

Ethics oversight | *Identify the organization(s) that approved or provided guidance on the study protocol, OR state that no ethical approval or guidance was required and explain why not.*

Note that full information on the approval of the study protocol must also be provided in the manuscript.

Human research participants

Policy information about studies involving human research participants

Population characteristics | A total of 67 patients with LS SCLC (11 females and 56 males with median age 64 (range: 38-82)) who underwent surgical resection at Zhejiang Cancer Hospital, Hangzhou, China from 2008 to 2020 were enrolled. All the enrolled patients underwent upfront surgery without preoperative chemotherapy or radiation and no patients received immunotherapy prior to or post surgery. As a control group, 68 patients with localized NSCLC (18 females and 50 males with median age 64 (range: 36-79)), who underwent upfront surgery without preoperative chemotherapy or radiation were included.

Recruitment | No patients were recruited specifically for this study. All patients were treated as standard of care and surgical specimens were randomly collected and analyzed. No self-selection process is involved.

Ethics oversight | Written informed consent was obtained from all patients involved. The study was approved by the Institutional Review Boards (IRB) at MD Anderson Cancer Center and Zhejiang Cancer Hospital. This study is compliant with the "Guidance of the Ministry of Science and Technology (MOST) for the Review and Approval of Human Genetic Resources", which requires formal approval for the export of human genetic material or data from China.

Note that full information on the approval of the study protocol must also be provided in the manuscript.

Clinical data

Policy information about clinical studies

All manuscripts should comply with the ICMJEguidelines for publication of clinical research and a completedCONSORT checklist must be included with all submissions.

Clinical trial registration | NCT01888601

Study protocol | https://www.nejm.org/doi/suppl/10.1056/NEJMoa1616288/suppl_file/nejmoa1616288_protocol.pdf

Data collection | Prospective

Outcomes | Intratumor heterogeneity mediated through chromosome instability was associated with an increased risk of recurrence or death.

Dual use research of concern

Policy information about dual use research of concern

Hazards

Could the accidental, deliberate or reckless misuse of agents or technologies generated in the work, or the application of information presented in the manuscript, pose a threat to:

No	Yes	
☐	☐	Public health
☐	☐	National security
☐	☐	Crops and/or livestock
☐	☐	Ecosystems
☐	☐	Any other significant area

Experiments of concern

Does the work involve any of these experiments of concern:

No	Yes	
☐	☐	Demonstrate how to render a vaccine ineffective
☐	☐	Confer resistance to therapeutically useful antibiotics or antiviral agents
☐	☐	Enhance the virulence of a pathogen or render a nonpathogen virulent
☐	☐	Increase transmissibility of a pathogen
☐	☐	Alter the host range of a pathogen
☐	☐	Enable evasion of diagnostic/detection modalities
☐	☐	Enable the weaponization of a biological agent or toxin
☐	☐	Any other potentially harmful combination of experiments and agents

ChIP-seq

Data deposition

☐ Confirm that both raw and final processed data have been deposited in a public database such as GEO.

☐ Confirm that you have deposited or provided access to graph files (e.g. BED files) for the called peaks.

Data access links<br>*May remain private before publication.*	*For "Initial submission" or "Revised version" documents, provide reviewer access links. For your "Final submission" document, provide a link to the deposited data.*
Files in database submission	*Provide a list of all files available in the database submission.*
Genome browser session<br>(e.g. UCSC)	*Provide a link to an anonymized genome browser session for "Initial submission" and "Revised version" documents only, to enable peer review. Write "no longer applicable" for "Final submission" documents.*

Methodology

Replicates	*Describe the experimental replicates, specifying number, type and replicate agreement.*
Sequencing depth	*Describe the sequencing depth for each experiment, providing the total number of reads, uniquely mapped reads, length of reads and whether they were paired- or single-end.*
Antibodies	*Describe the antibodies used for the ChIP-seq experiments; as applicable, provide supplier name, catalog number, clone name, and lot number.*
Peak calling parameters	*Specify the command line program and parameters used for read mapping and peak calling, including the ChIP, control and index files used.*
Data quality	*Describe the methods used to ensure data quality in full detail, including how many peaks are at FDR 5% and above 5-fold enrichment.*
Software	*Describe the software used to collect and analyze the ChIP-seq data. For custom code that has been deposited into a community repository, provide accession details.*

Flow Cytometry

Plots

Confirm that:

- ☐ The axis labels state the marker and fluorochrome used (e.g. CD4-FITC).
- ☐ The axis scales are clearly visible. Include numbers along axes only for bottom left plot of group (a 'group' is an analysis of identical markers).
- ☐ All plots are contour plots with outliers or pseudocolor plots.
- ☐ A numerical value for number of cells or percentage (with statistics) is provided.

Methodology

Sample preparation	*Describe the sample preparation, detailing the biological source of the cells and any tissue processing steps used.*
Instrument	*Identify the instrument used for data collection, specifying make and model number.*
Software	*Describe the software used to collect and analyze the flow cytometry data. For custom code that has been deposited into a community repository, provide accession details.*
Cell population abundance	*Describe the abundance of the relevant cell populations within post-sort fractions, providing details on the purity of the samples and how it was determined.*
Gating strategy	*Describe the gating strategy used for all relevant experiments, specifying the preliminary FSC/SSC gates of the starting cell population, indicating where boundaries between "positive" and "negative" staining cell populations are defined.*

- ☐ Tick this box to confirm that a figure exemplifying the gating strategy is provided in the Supplementary Information.

Magnetic resonance imaging

Experimental design

Design type	*Indicate task or resting state; event-related or block design.*
Design specifications	*Specify the number of blocks, trials or experimental units per session and/or subject, and specify the length of each trial or block (if trials are blocked) and interval between trials.*
Behavioral performance measures	*State number and/or type of variables recorded (e.g. correct button press, response time) and what statistics were used to establish that the subjects were performing the task as expected (e.g. mean, range, and/or standard deviation across subjects).*

Acquisition

Imaging type(s)	*Specify: functional, structural, diffusion, perfusion.*
Field strength	*Specify in Tesla*
Sequence & imaging parameters	*Specify the pulse sequence type (gradient echo, spin echo, etc.), imaging type (EPI, spiral, etc.), field of view, matrix size, slice thickness, orientation and TE/TR/flip angle.*
Area of acquisition	*State whether a whole brain scan was used OR define the area of acquisition, describing how the region was determined.*

Diffusion MRI ☐ Used ☐ Not used

Preprocessing

Preprocessing software	*Provide detail on software version and revision number and on specific parameters (model/functions, brain extraction, segmentation, smoothing kernel size, etc.).*
Normalization	*If data were normalized/standardized, describe the approach(es): specify linear or non-linear and define image types used for transformation OR indicate that data were not normalized and explain rationale for lack of normalization.*
Normalization template	*Describe the template used for normalization/transformation, specifying subject space or group standardized space (e.g. original Talairach, MNI305, ICBM152) OR indicate that the data were not normalized.*
Noise and artifact removal	*Describe your procedure(s) for artifact and structured noise removal, specifying motion parameters, tissue signals and physiological signals (heart rate, respiration).*

Volume censoring | *Define your software and/or method and criteria for volume censoring, and state the extent of such censoring.*

Statistical modeling & inference

Model type and settings | *Specify type (mass univariate, multivariate, RSA, predictive, etc.) and describe essential details of the model at the first and second levels (e.g. fixed, random or mixed effects; drift or auto-correlation).*

Effect(s) tested | *Define precise effect in terms of the task or stimulus conditions instead of psychological concepts and indicate whether ANOVA or factorial designs were used.*

Specify type of analysis: ☐ Whole brain ☐ ROI-based ☐ Both

Statistic type for inference (See Eklund et al. 2016) | *Specify voxel-wise or cluster-wise and report all relevant parameters for cluster-wise methods.*

Correction | *Describe the type of correction and how it is obtained for multiple comparisons (e.g. FWE, FDR, permutation or Monte Carlo).*

Models & analysis

n/a	Involved in the study
☐	☐ Functional and/or effective connectivity
☐	☐ Graph analysis
☐	☐ Multivariate modeling or predictive analysis

Functional and/or effective connectivity | *Report the measures of dependence used and the model details (e.g. Pearson correlation, partial correlation, mutual information).*

Graph analysis | *Report the dependent variable and connectivity measure, specifying weighted graph or binarized graph, subject- or group-level, and the global and/or node summaries used (e.g. clustering coefficient, efficiency, etc.).*

Multivariate modeling and predictive analysis | *Specify independent variables, features extraction and dimension reduction, model, training and evaluation metrics.*
